# Convolutional neural networks in brain disease diagnosis: a unified review of Alzheimer's, Parkinson's, and brain tumor classification

**DOI:** 10.3389/fnins.2026.1875642

**Published:** 2026-07-29

**Authors:** R. Nathea, Kalyanbrata Ghosh, J. S. Nisha

**Affiliations:** 1 School of Electronics Engineering, Vellore Institute of Technology, Vellore, Tamil Nadu, India; 2 Computer Science and Engineering, Indian Institute of Information Technology, Kottayam, Kerala, India

**Keywords:** Alzheimer's disease, brain disorders, brain tumor, convolutional neural networks, magnetic resonance imaging, Parkinson's disease

## Abstract

Neurological and neuro-oncological brain disorders like Alzheimer's disease (AD), Parkinson's disease (PD), and brain tumors are challenging to diagnose due to overlapping symptoms and the limitations of conventional imaging techniques. Magnetic resonance imaging (MRI) with convolutional neural networks (CNNs) has emerged as a powerful approach, enabling automated and high-precision detection and staging. This review critically integrates recent developments in CNN architectures, such as hybrid models, attention mechanisms, and 3D CNNs for MRI-based diagnosis of these disorders. It further examines preprocessing methods, datasets, and performance metrics across studies, with emphasis on innovations such as transformer-based models and lightweight architectures. While CNNs show impressive accuracy, issues remain in generalizability, interpretability, and clinical integration. This review highlights the need for multimodal data fusion, explainable artificial intelligence, and real-world validation to narrow the gap between research and clinical practice. By defining future directions, this review aims to guide the development of robust, scalable neurodiagnostic systems for early intervention and better patient outcomes.

## 1 Introduction

Neurological and neuro-oncological brain disorders continue to represent a serious global healthcare concern. They impact millions of people globally and are becoming a growing burden on healthcare systems. AD, PD, and various brain tumors are more common among these diseases owing to their frequency, complex progression, and high rates of disability and death (Khan et al., 2024; Hannan, 2024; Belhadi et al., 2025). Effective management of these diseases depends heavily on early, precise diagnosis (Dentamaro et al., 2024). Traditional diagnostic tests often fail to detect these diseases at an early stage. Consequently, computational models have attracted increased interest (Turrisi et al., 2025). These models seek to enhance diagnostic accuracy and assist clinical decision-making. Through this, they provide a more subtle and advanced way of identifying and classifying illness.

AD is the most prevalent form of dementia, which is manifested by progressive neurodegeneration and results in impaired ability to perform daily functions along with diminishing cognition and memory (Mahjoubi et al., 2025). According to the World Health Organization, the cases are set to exceed cancer in 2050 and stand at a rate of 152 million (Khatri and Kwon, 2024). AD is one of the primary causes of death globally. Pathologically, it is characterized by amyloid-beta plaque and tangles of tau proteins that injure neurons and cause brain atrophy (Ghadami and Rahebi, 2025). Such pathologic defects target the hippocampus as well as the cortices before ultimately causing irreversible cognition damage. As AD is incurable, early diagnosis, particularly at the stage of mild cognitive impairment (MCI), is most critical for effective treatment to retard disease progress and improve quality of life (Chen et al., 2024).

PD is caused by the neuronal degeneration in the substantia nigra damaging dopaminergic pathways (Tassew et al., 2023). Bradykinesia, tremors, and disrupted gait are the initial symptoms of the disease (Hussain et al., 2023). This is followed by impairments in executive functioning, visuospatial abilities, and memory. Diagnosis is made through clinical examination, neuroimaging, and biomarker assessment, but it is not possible in the initial stage as features overlap with other neurologic diseases (Kanagaraj et al., 2024). Neuroimaging techniques have established PD-related neurodegeneration, but the very high false-positive rate during the early detection period emphasizes the need for advanced deep learning (DL) based diagnostic techniques (Reddy et al., 2024).

Brain tumors, that is, unrestrained proliferations of brain tissue, continue to be one of the most fatal types of cancer (Md. Ashafuddula and Islam, 2024). Brain tumors result in serious neurological impairment like headaches, seizures, loss of hearing and sight, and intellectual decline (Srinivasan et al., 2024). Symptom severity is a function of tumor location and rate of growth, with high growth rate aggressive tumors quickly worsening neurological function (Rabby et al., 2024). Though glioma, meningioma, and pituitary tumors seem unique on MRI, minor structural differences make accurate classification challenging. Early detection is vital for enhancing survival since the effectiveness of treatment relies directly on the size and malignancy of tumors at diagnosis (Qureshi et al., 2025). Traditional diagnosis techniques are thwarted by heterogeneity in brain tumors (Zhu et al., 2024). Hence, effective, automated algorithms need to be formulated for accurate classification and prognosis.

MRI is a critical non-invasive diagnostic tool for neurological and neuro-oncological brain disorders (Rezk et al., 2025). In comparison to other imaging techniques like computed tomography (CT), positron emission tomography (PET), and single-photon emission computed tomography (SPECT) which use ionizing radiation, MRI is particularly sensitive to alterations in brain function and structure without subjecting the patients to exposure of ionizing radiation (Simo et al., 2024).

To quantify AD progression, marker levels such as cerebrospinal fluid, cortical thickness, and gray and white matter volumes are helpful (Momeni et al., 2025). Analysis of MRI has succeeded in showing structural changes in PD in relation to dopamine deficiency and facilitates disease staging and differentiation from other movement disorders (Suwalska et al., 2023). MRI is viewed as a golden standard in the examination of the structure and development of brain tumors (Al-Otaibi et al., 2024). MRI sequences like T1-weighted and T2-weighted have proved significant while quantifying disease-associated change in the brain (Grødem et al., 2024). In some instances, it is also used alongside other modalities such as PET, SPECT, clinical information, and genetic information (Castellano et al., 2024).

Routine MRI-based diagnosis is largely dependent on interpretation by radiologists, a step that is time-consuming, subject to inter-observer variability, and usually insufficient in identifying subtle, early-stage pathological changes (Muksimova et al., 2025). While automatic classification has been performed using traditional machine learning algorithms such as support vector machines, random forests, and k-nearest neighbors, their reliance on manually created features hinders them from scaling and capturing completely the complex patterns involved in MRI data (Rasa et al., 2024). On the other hand, more recent advances in DL, particularly CNNs, have substantially improved diagnostic accuracy by enabling hierarchical automatic extraction of features from imaging data (Liao et al., 2024). These models are capable of identifying intricate and often undetectable structural changes and are therefore less sensitive to variation in imaging modalities and less dependent on human intervention (Turk et al., 2024; Ben Brahim et al., 2024). As a result, CNNs have been shown to have great promise in facilitating early diagnosis, influencing treatment, and facilitating longitudinal observation of neurodegenerative and oncological brain disorders (Özaltın, 2025; Onaizah et al., 2025).

Alzheimer's disease (AD), Parkinson's disease (PD), and brain tumors were selected for this review due to their high global prevalence, mortality burden, and clinical relevance in neuroimaging research. The World Health Organization (WHO) estimates that approximately 57 million people worldwide were living with dementia in 2021, with Alzheimer's disease accounting for 60%–70% of dementia cases. Parkinson's disease currently affects more than 10 million people globally. Neurological disorders collectively affect more than 3 billion individuals and contribute to nearly 11 million deaths each year worldwide. Brain and central nervous system tumors also represent a major clinical challenge, with more than 300,000 new cases reported globally each year. These disorders are associated with progressive cognitive, motor, and functional impairment, for which early diagnosis is essential to improve treatment outcomes and patient survival. The availability of public MRI datasets and the increasing demand for automated diagnostic tools have contributed to extensive research using convolutional neural network (CNN)-based deep learning methods in neuroimaging applications involving these diseases.

Unlike prior reviews that focused primarily on a single neurological disorder or a specific deep learning architecture, this review provides a unified comparative analysis of CNN-based approaches for Alzheimer's disease, Parkinson's disease, and brain tumor diagnosis using MRI data. This review highlights the architectures and performance of the latest CNN models as well as identifies and discusses the potential limitations of the models, such as dataset dependence, processing variability, validation techniques, model generalizability, clinical relevance, and ethical aspects for various neurological disorders. Moreover, the study identifies evolving trends in the field, including hybrid CNN-TF approaches, light architectures, explainable AI (XAI), and multimodal learning frameworks, and uncovers critical challenges that persist in the path toward clinical translation in the real world.

A systematic review was performed according to PRISMA guidelines to find recent studies that used CNN for the diagnosis of brain disorders from MRI. We searched the widely used databases PubMed, ScienceDirect, IEEE Xplore, Google Scholar, and SpringerLink. The literature search covered studies published between January 2020 and March 2025 using combinations of keywords such as (“Alzheimer's disease” OR “Parkinson's disease” OR “brain tumor” OR “glioma”) AND (“MRI” OR “magnetic resonance imaging”) AND (“CNN” OR “convolutional neural network”) AND (“diagnosis” OR “classification”). This search identified 2,665 records, with 2,165 remaining after removing duplicates. Following title and abstract screening, 165 articles were assessed for full-text eligibility. Based on predefined inclusion criteria—(i) MRI-based CNN studies, (ii) peer-reviewed English articles, and (iii) studies reporting quantitative performance metrics—98 studies were finally included in the review. Review articles, duplicate records, non-MRI-based studies, conference abstracts, and studies lacking sufficient methodological or validation details were excluded.

Among these, 60 studies (20 each for Alzheimer's disease, Parkinson's disease, and brain tumor classification) were selected for detailed comparative analysis to ensure balanced representation and methodological consistency across datasets, evaluation metrics, and experimental settings. Studies lacking sufficient methodological detail or standardized evaluation metrics were not included in the detailed comparative analysis but were considered in the broader discussion of datasets, preprocessing techniques, segmentation approaches, limitations, and future research directions.

**Figure 1:**
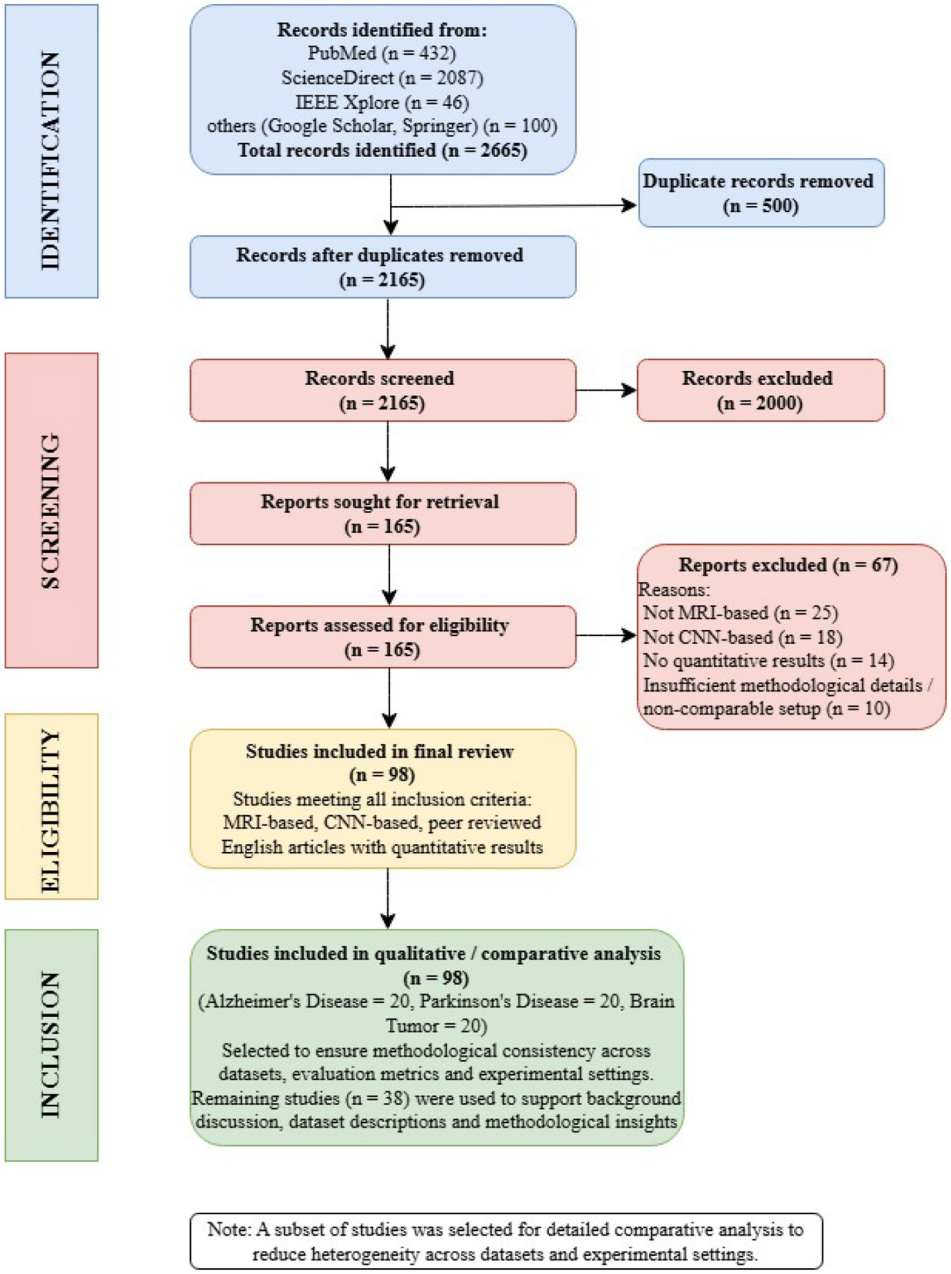
PRISMA flowchart depicting the search strategy.

For the detailed comparative analysis, each included study was systematically evaluated using five predefined methodological quality criteria: (i) independent external validation, (ii) patient-level data separation, (iii) explainable artificial intelligence, (iv) statistical validation, and (v) computational efficiency reporting. These criteria were selected to evaluate methodological rigor, reproducibility, interpretability, and clinical applicability across CNN-based studies on neurological and neuro-oncological brain disorders. Although this structured methodological quality assessment provides a consistent framework for comparative evaluation, it was designed to assess methodological quality and should not be interpreted as a formal standardized risk-of-bias assessment. The detailed study selection process is illustrated in Figure 1. This review focuses on presenting an overview of the existing literature on the utilization of DL methods, with emphasis on models based on the neural network architecture, for AD, PD, and brain tumor diagnosis and classification from MRI data. Through the analysis of cutting-edge methodologies, this research will evaluate the strengths and weaknesses of different architectures, address current challenges in the area, and identify possible future directions for incorporating artificial intelligence (AI) into the management of these life-threatening neurological disorders.

## 2 Deep learning architectures for neuroimaging analysis

CNNs are a cornerstone of DL models applied in medical imaging. CNNs process data with a grid-like topology, which is ideal for analyzing images by learning spatial hierarchies through layers of convolutional filters (Yakkundi et al., 2024). They automatically learn features such as edges, textures, and complex patterns. CNNs usually comprise convolutional layers, non-linear activations (ReLU), pooling layers, and fully connected layers for final classification (Bai et al., 2024). 3D CNNs generalize standard CNNs to process volumetric data, like MRI scans. An exemplary architecture could consist of several 3D convolutional and pooling layers, dropout regularization, batch normalization for training stability, and dense layers using sigmoid activation for binary classification. 3D CNNs are well-suited for modeling spatial relationships in three dimensions, which is important in neuroimaging (Priyadharshini et al., 2024).

Transfer learning (TL) has commonly been utilized to address the data shortage in medical imaging. Fine-tuning a model initially trained on a large dataset such as ImageNet is done to specialize it for the particular neuroimaging task (Rehman et al., 2024). This reduces training time and enhances performance. Several well-established CNN architectures have been adapted for neuroimaging:

- VGG simplifies model design by using uniform 3 × 3 convolutional kernels and deep networks. It is used as a standard reference in numerous medical imaging applications.
- EfficientNet achieves balance between accuracy and computational cost by compound scaling of depth, width and resolution.
- Inception adds parallel convolutions having different kernel sizes, enabling multi-scale feature extraction in a single layer.
- ResNet solves the vanishing gradient problem using residual connections, enabling the training of very deep networks.
- DenseNet encourages feature reuse by connecting each layer to every other layer, improves gradient flow, and reduces the number of parameters.
- Xception replaces standard convolutions with depthwise separable convolutions, improving computational efficiency while maintaining accuracy.

U-Net is a convolutional neural network (CNN) architecture widely used for medical image segmentation. Its encoder-decoder design with skip connections supports accurate localization, making it well-suited for tasks such as brain tumor segmentation. MobileNet uses depthwise separable convolutions to achieve efficient inference with a reduced number of parameters, making it suitable as a lightweight backbone for classification and object detection pipelines.

Transformers, originally developed for natural language processing, are adopted in neuroimaging due to their ability to model global dependencies. They are renowned for their self-attention mechanisms that capture long-range dependencies and have been incorporated into CNNs (Özdemir and Özyurt, 2025). In 3D contexts, hybrid methods are more desirable, such as the embedding of CNN modules into transformers or transformers into CNN, as they are expensive. Spatially separable convolutions can be used as an efficient alternative to full self-attention while maintaining the global context with reduced computation cost (Pacal, 2024).

CNN-RNN hybrid networks, including long short-term memory (LSTM), have also been developed. RNNs capture temporal patterns for longitudinal and time-series fMRI analysis, while CNNs extract spatial features. Hybrid CNN-graph neural networks integrate spatial information and ROI connectivity. CNN-autoencoder combinations enable unsupervised feature learning, dimensionality reduction, denoising, and compression of high-dimensional neuroimaging data for classification.

### 2.1 Performance measures

The comparison of CNN models in neuroimaging is often performed based on four primary performance measures: accuracy, precision, recall, and F1-score Awang et al. (2024). These evaluation metrics are defined in Equations 1–4. Accuracy (Acc) calculates the overall classification accuracy of the model across all classes.


$$\begin{array}{l} \text{Acc}=\frac{TP+TN}{TP+TN+FP+FN} \end{array}$$ (1)

Precision (Prec) indicates the number of correct predictions of positive instances out of total positive predictions.


$$\begin{array}{l} \text{Prec}=\frac{TP}{TP+FP} \end{array}$$ (2)

Recall (Rec) refers to the potential of the model to capture all true positive cases.


$$\begin{array}{l} \text{Rec}=\frac{TP}{TP+FN} \end{array}$$ (3)

F1-Score (F1) offers a trade-off between recall and precision and is particularly valuable in class imbalance situations.


$$\begin{array}{l} F_{1}=2\times \frac{\text{Prec}\times \text{Rec}}{\text{Prec}+\text{Rec}} \end{array}$$ (4)

These measures are commonly used in the reviewed studies, as they are pertinent for evaluating diagnostic performance in medical contexts.

## 3 CNN in AD diagnosis and classification

### 3.1 Datasets

**Figure 2:**
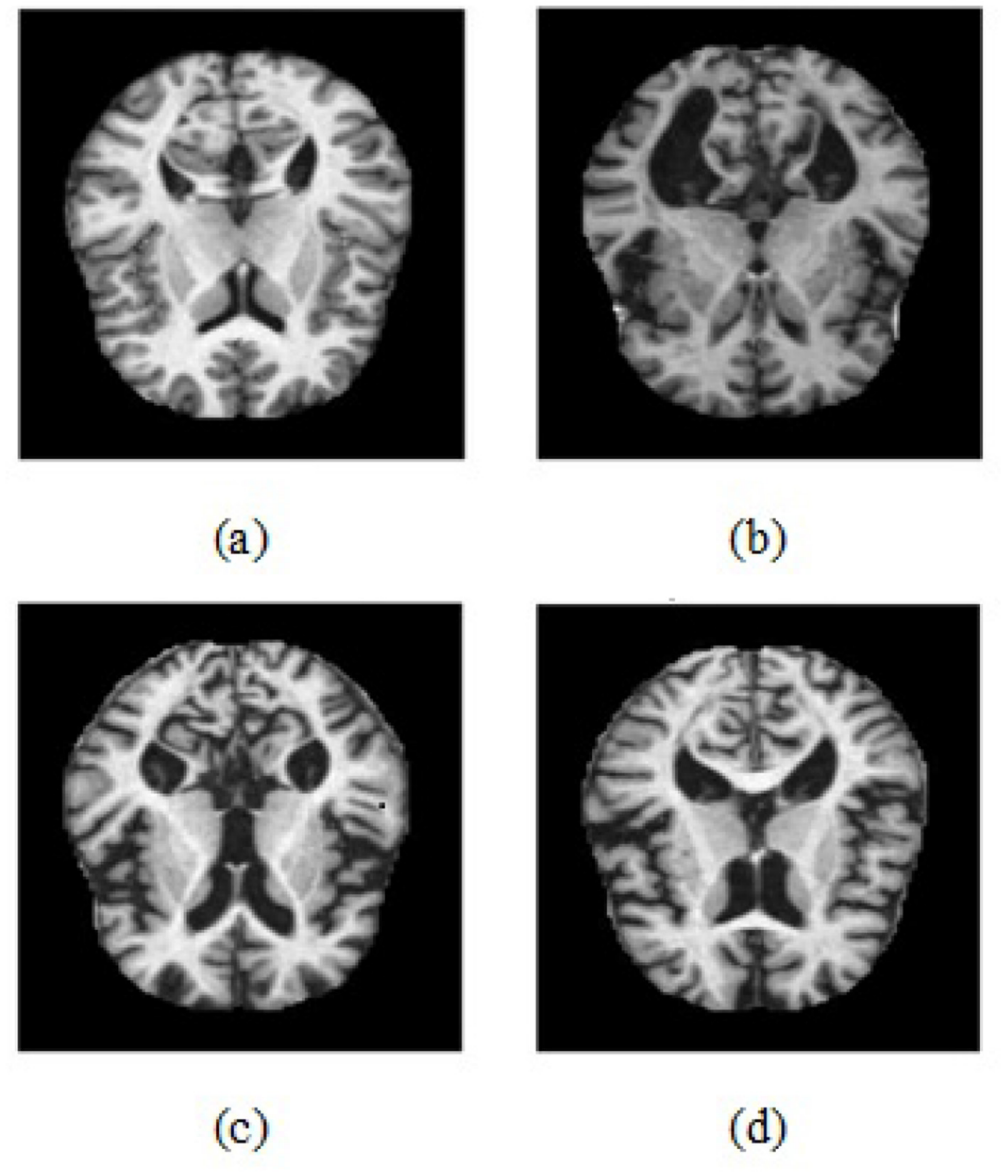
MRI images of AD: **(a)** non-demented (ND), **(b)** mildly demented (MID), **(c)** moderately demented (MOD), **(d)** very mildly demented (VMD).

**Figure 3:**
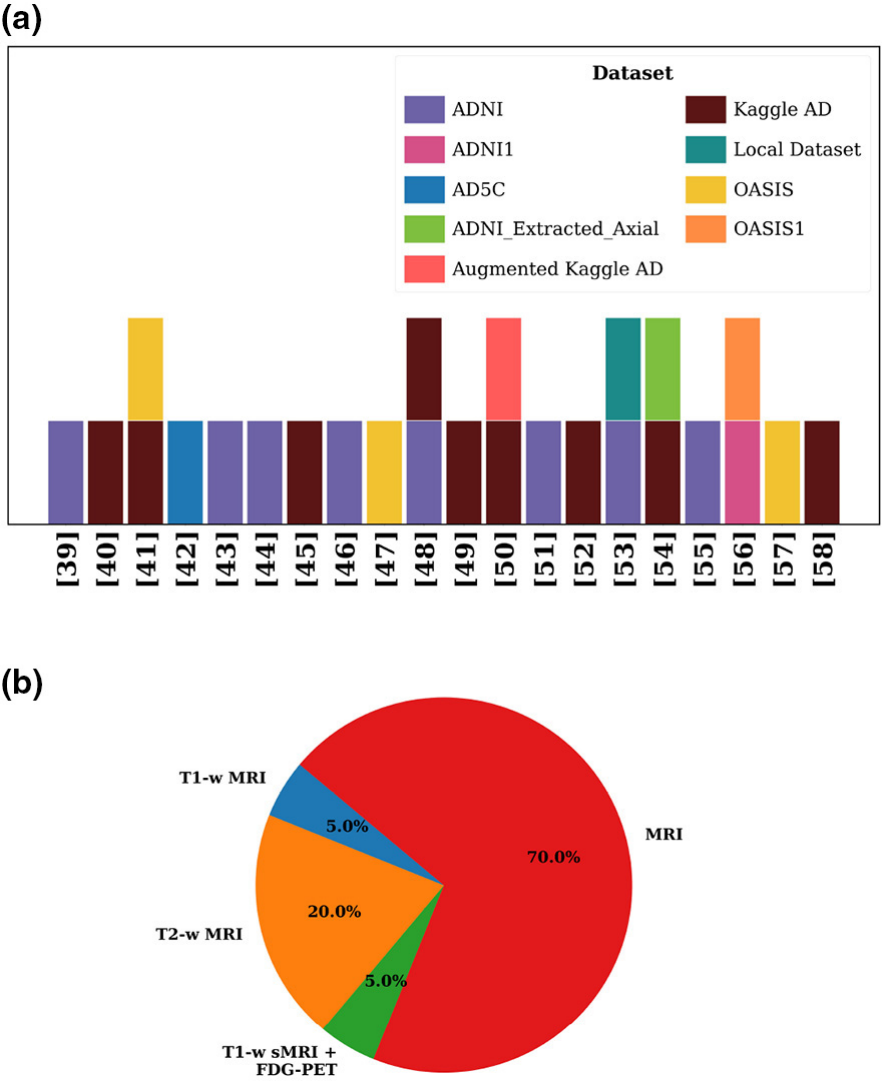
Dataset and imaging modality distribution in CNN-based Alzheimer's disease studies: **(a)** dataset usage across reviewed studies, **(b)** image modality distribution in AD studies.

The samples of the four classes of AD patients are depicted in Figure 2. The datasets frequently utilized for the analysis of AD are the Alzheimer's Disease Neuroimaging Initiative (ADNI), Kaggle (4 classes of images), open access series of imaging studies (OASIS), and AD5C. The dataset usage across reviewed studies and Image modality distribution in AD studies are illustrated in Figures 3a, b. The database of ADNI includes a diverse set of AD data, such as MRI, PET, clinical assessment data, and genetic data. It includes longitudinal MRI that assists in disease progression classification (Awarayi et al., 2024). The Kaggle AD dataset contains 6,400 MRI images that are labeled non-demented, mild demented, moderate demented, and very mild demented (Slimi et al., 2024). The OASIS database includes various datasets such as longitudinal MRI, cross-sectional MRI, clinical data, and longitudinal multimodal data (Tripathy et al., 2024). The AD5C dataset from the Kaggle database contains 2,380 MRI images that are labeled into five phases of the disease (Awang et al., 2024). Table 1 summarizes the datasets, modalities, dataset sizes, classification categories, validation strategies, and dataset availability employed in the reviewed AD based studies.

**Table 1:** Summary of datasets, modalities, validation methods, and dataset availability employed in reviewed AD classification studies.

References	Dataset/modality	Dataset size	Classes	Validation method	Dataset availability
Awarayi et al. (2024)	ADNI (MRI)	4,482 images: 1,581 AD, 1,310 MCI, and 1,591 NC	AD/MCI/NC (3-class)	10-fold cross-validation	https://adni.loni.usc.edu
Slimi et al. (2024)	Kaggle Alzheimer MRI dataset (MRI)	>5,000 images	VMD/MID/MOD/ND (4-class)	80:20 train-test split with 5-fold cross-validation	https://www.kaggle.com/datasets/tourist55/alzheimers-dataset-4-class-of-images
Tripathy et al. (2024)	Kaggle Alzheimer MRI dataset (MRI); OASIS dataset (MRI)	5,600 MRI scans (Kaggle dataset); 399 subjects (OASIS dataset)	VMD/MID/MOD/ND (4-class)	90:10 train-test split (Kaggle dataset); 2-fold cross-validation (OASIS-1 dataset)	https://www.kaggle.com, https://oasis-brains.org
Awang et al. (2024)	AD5C Kaggle dataset (MRI)	2,381 MRI images	VMD/MID/MOD/ND/SD(Severe Demented) (5-class)	80:10:10 (Train:Validation:Test)	https://www.kaggle.com
Tang et al. (2024)	ADNI multimodal dataset (sMRI + 18F-FDG PET)	210 subjects: 88 AD, 122 CN	AD/CN (binary classification)	10-fold cross-validation	https://adni.loni.usc.edu
Nithya et al. (2024)	ADNI1 dataset (MRI)	1,726 MRI scans from 479 subjects	AD vs. non-AD binary classification	Training-validation strategy reported	https://adni.loni.usc.edu
Mahanty et al. (2024)	Kaggle Alzheimer MRI dataset (MRI)	6,400 MRI scans	VMD/MID/MOD/ND (4-class)	80:20 train-test split with augmentation	https://www.kaggle.com/datasets/tourist55/alzheimers-dataset-4-class-of-images
Rahman et al. (2025)	ADNI dataset (T1-weighted 3D MRI scans)	2,182 MRI scans from 221 subjects	CN/MCI/AD (3-class)	70% training, 15% validation, and 15% testing split	https://adni.loni.usc.edu
Khaleel and Lakizadeh (2025)	OASIS dataset (MRI)	5,692 MRI images	VMD/MID/MOD/ND (4-class)	Not explicitly reported	https://oasis-brains.org
Sasithradevi et al. (2024)	ADNI dataset (MRI) and Kaggle dataset (MRI)	1,101 MRI images (ADNI); 6400 MRI images (Kaggle)	AD/LMCI/MCI/EMCI/CN (5-class) and VMD/MID/MOD/ND (4-class)	80% training and 20% testing split	https://adni.loni.usc.edu/data-samples, https://www.kaggle.com
Sorour et al. (2024)	Kaggle dataset (MRI)	6,400 MRI images	Demented/non-demented (binary classification)	80% training and 20% testing split	https://www.kaggle.com/datasets/tourist55/alzheimers-dataset-4-class-of-images
Tuncer et al. (2025)	Kaggle dataset (original images) + augmented Alzheimer MRI dataset (MRI)	40,384 MRI images	VMD/MID/MOD/ND (4-class)	70:30 training-validation split; testing on original images	https://www.kaggle.com/datasets/uraninjo/augmented-alzheimer-mri-dataset, https://www.kaggle.com/datasets/uraninjo/augmented-alzheimer-mri-dataset-v2
El-Assy et al. (2024)	ADNI dataset (MRI)	1,296 T1-weighted 3D MRI scans	AD/LMCI/MCI/EMCI/CN (5-class)	90% training data with 90:10 training-validation split and 10% testing data	https://adni.loni.usc.edu
Ali et al. (2024a)	Kaggle Alzheimer's Dataset (MRI)	5,121 MRI slices	VMD/MID/MOD/ND (4-class)	Random 80:20 training-testing split with augmentation	https://www.kaggle.com/datasets/tourist55/alzheimers-dataset-4-class-of-images
Fathi et al. (2024)	ADNI dataset (T2-weighted MRI); local dataset from Firoozgar Hospital (T2-weighted MRI)	14,241 MRI slices (ADNI); 238 MRI slices (Local dataset)	AD/LMCI/MCI/EMCI/NC (5-class) and AD/NC/MCI (3-Class)	ADNI training with independent local external validation	https://adni.loni.usc.edu
Ali et al. (2024b)	Alzheimer's Dataset (MRI); ADNI_Extracted_Axial Dataset (MRI)	5,121 MRI slices; 5,154 MRI slices	MID/MOD/VMD/ND (4-class) and MID/MOD/VMD (3-class)	80:20 split with additional 10-fold cross-validation	https://www.kaggle.com/datasets/tourist55/alzheimers-dataset-4-class-of-images, https://www.kaggle.com/datasets/katalniraj/adniextracted-axial
Zhao et al. (2024)	ADNI dataset (MRI)	2,248 T1-weighted 3D MRI scans from 818 subjects	AD/MCI/NC (3-class)	8:1:1 training-validation-testing split with subject-level 10-fold cross-validation	https://adni.loni.usc.edu
Hassan et al. (2025)	ADNI1 1-Year 1.5T dataset (MRI); Kaggle dataset (MRI); OASIS-1 dataset (MRI)	2,294 MRI scans; 6,400 MRI images; 9,600 MRI images	AD/MCI/NC (3-class) and VMD/MID/MOD/ND (4-class)	60% training, 20% validation, and 20% testing split	https://adni.loni.usc.edu, https://www.kaggle.com, https://oasis-brains.org
Yan et al. (2025)	OASIS dataset (MRI)	6,400 T1-weighted MRI images	Non-Demented/Demented (binary classification)	70% training, 10% validation, and 20% testing split	https://oasis-brains.org
Asaduzzaman et al. (2025)	Kaggle MRI dataset (MRI)	6,400 MRI images	VMD/MID/MOD/ND (4-class)	80:20 split with SMOTE-based class balancing	https://www.kaggle.com

### 3.2 Preprocessing and data augmentation

Common preprocessing techniques used in MRI-based AD classification included image conversion, resizing, skull stripping, normalization, bias field correction, filtering, and contrast enhancement (Tang et al., 2024; Nithya et al., 2024). Several studies additionally employed augmentation and balancing strategies such as rotation, flipping, SMOTE, and ADASYN to reduce overfitting and address class imbalance (Slimi et al., 2024; El-Assy et al., 2024; Asaduzzaman et al., 2025).

### 3.3 Segmentation

Segmentation plays a pivotal role in isolating regions of interest such as the hippocampus, a key biomarker in AD, from pre-processed MRI scans. A K-means clustering algorithm was utilized to partition MRI data into k distinct groups based on Euclidean distances to centroids, optimizing intra-cluster similarity (Nithya et al., 2024). To enhance brain region segmentation, morphological operations such as erosion and dilation were applied to refine the binary images (Rahman et al., 2025). A U-Net model, either pre-trained or custom-trained, was employed to accurately isolate brain structures using its deep learning-based feature extraction capabilities (Khaleel and Lakizadeh, 2025).

### 3.4 CNN models

A wide range of CNN-based architectures, including AlexNet, VGG16, ResNet, and DenseNet, as well as modern transfer learning (TL) approaches utilizing EfficientNet, Inception, and Xception, have been employed for AD classification (Sasithradevi et al., 2024). Several studies also implemented customized CNN variants such as depth-separable CNNs, dual-path CNNs, hybrid CNN-LSTM and CNN-transformer frameworks, and ensemble approaches integrating multiple pre-trained networks (Sorour et al., 2024). Besides, various advanced attention mechanisms, spatial filtering strategies, and lightweight architectures like FiboNeXt were explored to enhance classification accuracy and efficiency (Tuncer et al., 2025). The shift from conventional CNNs to hybrid and attention-based architectures underscores the need to capture complex spatial and temporal patterns in neuroimaging data, strengthening feature representations and improving diagnostic accuracy. Table 2 summarizes the different CNN models used for AD classification and their performance across different studies.

**Table 2:** Summary of CNN-based DL studies for AD classification using MRI data.

References	Data preparation	Model	Performance metrics	Architectural trade-offs and limitations
Awarayi et al. (2024)	Image conversion, histogram equalization, bilateral filtering	4-layer CNN, ReLU, dropout	MCI vs. Normal controls, Multiclass Acc: 95.62, 93.45 Prec 95.63, 93.70 Rec: 95.64, 93.24	Bilateral filtering with a lightweight CNN improved noise-robust feature learning, but aggressive image resizing may reduce fine anatomical detail preservation.
Slimi et al. (2024)	Resizing, augmentation, balancing (SMOTE)	Hybrid Model: DenseNet121 + Xception	Acc/Prec/F1: 99.85; Rec: 99.88	Hybrid DenseNet121-Xception fusion improved feature representation and noise robustness, but increased architectural complexity and computational cost.
Tripathy et al. (2024)	Not specified	Depth-separable CNN (DSC) with spatial attention	Kaggle, OASIS Acc: 96.25, 99.75 Prec: 96.71, 99.63 Rec: 96.36, 99.91 F1: 96.52, 99.77	Multiscale spatial attention improved discriminative feature representation, but external generalization capability was limited.
Awang et al. (2024)	Resizing, augmentation	Deep TL with DenseNet-201	Acc: 98.24 Prec: 97.8 Rec: 97.6 F1: 98.2	DenseNet-201 transfer learning improved feature reuse and classification accuracy, but performance depended heavily on augmentation-based data balancing.
Tang et al. (2024)	AC-PC reorientation, bias correction, skull stripping, cerebellum removal	Improved transformer on 3D CNN features followed by MLP classification	Acc: 98.1 Prec: 99.09 Rec: 95.82 F1: 97.81	Gradual self-attention activation improved training stability, but delayed global-context learning during early optimization.
Nithya et al. (2024)	SMOTE, CLAHE, BADF, ICP	ResNet-50 with shortcut links in residual layers	Acc: 95.9 Prec: 95 Rec: 89 F1: 92.4	Sequential preprocessing improved MRI standardization, but cumulative preprocessing errors could affect downstream classification robustness.
Mahanty et al. (2024)	Bias field correction, noise removal, RGB normalization, resizing, augmentation	Enhanced Xception ensemble with SE blocks, dilated convolutions, FPN, and random forest blending.	Acc: 99.14 Prec: 93.82 Rec: 97.17 F1: 95.37	Snapshot-based enhanced Xception improved multi-scale feature learning and generalization, but progressive ensemble augmentation increased optimization and inference complexity.
Rahman et al. (2025)	Skull stripping, spatial normalization, smoothing, frame selection, cropping	3D-CNN with 3D convolutions, batch normalization, ReLU, max-pooling, FC, and softmax.	Acc: 92.89 Prec: 91.99 Rec: 92.54 F1: 92.21	Intelligent frame selection and 3D dilated convolutions improved volumetric feature learning with fewer parameters, but model performance remained dependent on preprocessing quality.
Khaleel and Lakizadeh (2025)	Skull stripping, Gaussian blur, Otsu's thresholding, normalization, augmentation	ResNet-50 + Fully stacked bidirectional LSTM (FSBi-LSTM)	Acc: 99.6 Prec: 99.6 Rec: 97.3 F1: 97.7	ResNet50-FSBiLSTM fusion improved spatial–contextual feature learning, but heavy preprocessing and class imbalance could affect robustness and generalizability.
Sasithradevi et al. (2024)	Resizing, augmentation	CNN: AlexNet, GoogLeNet, VGG16, ResNet, EfficientNet, DenseNet	Acc/ Prec/ Rec/ F1: ADNI: 99 Kaggle: 98.1	Hybrid handcrafted-deep feature fusion improved complementary feature representation, but ALO-based optimization introduced feature-selection dependency and high-dimensional redundancy.
Sorour et al. (2024)	Standardization, normalization, augmentation	CNN (spatial), LSTM (temporal), VGG16 (TL)	Acc: 99.2 Prec: 100 Rec: 99.5 F1: 99.7	CNN-LSTM and CNN-SVM fusion improved feature learning and decision boundaries, but augmentation-dependent training and binary class simplification could limit clinical generalizability.
Tuncer et al. (2025)	Not specified	FiboNeXt: lightweight CNN inspired by the Fibonacci sequence	AD, AD v2 Acc: 99.66, 99.63 Prec: 99.75, 99.69 Rec: 99.81, 99.81 F1: 99.78, 99.75	Fibonacci-inspired depth concatenation and attention blocks improved parameter-efficient feature learning, but augmented-data dependency and complex block design could limit cross-dataset robustness.
El-Assy et al. (2024)	Scaling, augmentation, balancing (ADASYN)	Dual-path CNN with progressive filtering	Acc/ Prec/ Rec: 3-way: 99.43 4-way: 99.57 5-way: 99.30	Dual-CNN feature concatenation improved multiclass classification, but the 57.5M-parameter architecture and augmentation-dependent training may limit generalizability.
Ali et al. (2024a)	Augmentation	Canonical correlation approach merged deep features from EfficientNet-b0 and MobileNet-v2	Acc: CCA-94.7 WOA-98.25	CCA-based deep feature fusion improved complementary representation learning, but optimization-driven feature selection increased pipeline dependency and computational overhead.
Fathi et al. (2024)	Normalization, resizing, removal of non-informative slices	Weighted probability-based ensemble method: DenseNet201 + DenseNet169 + DenseNet121 + ResNet50 + Inception-ResNet V2 + VGG19	LMCI vs. AD, 4-way Acc: 99.83, 93.88 Prec: 99.7, 94.08	WPBEM improved classification robustness, but combining six CNNs increased computational overhead, while the 2D slice-based strategy limited full volumetric feature representation.
Ali et al. (2024b)	Cropping, zero-centering, normalization	Stage 1 (Binary): 26-layer CNN; Stage 2 (Subclassification): TL	Acc/ Prec/ Rec F1: Binary: 98.24 Multiclass: 99.70	Multistage transfer-learning CNN improved dementia subclassification with reduced training time, but dependence on single-modality MRI and staged learning could limit robustness across heterogeneous datasets.
Zhao et al. (2024)	Orientation, spatial registration, skull stripping, bias field correction, enhancement, normalization	Hybrid VECNN (modified ResNet-50 + ViT-inspired elements)	Acc: 92.14 Prec: 86.84 Rec: 93.27	VECNN improved global contextual learning through ViT-inspired 3D feature extraction, but extensive preprocessing and modified ResNet-based design increased pipeline dependency and optimization complexity.
Hassan et al. (2025)	Image conversion, SMOTETomek	SCCAN (Stacked CNN + channel attention network)	Acc: ADNI—99.58 Kaggle—99.22 OASIS—99.70	Stacked CNN and channel attention improved hierarchical feature learning, but preprocessing-dependent training and SMOTETOMEK-based balancing may limit cross-dataset generalizability.
Yan et al. (2025)	Format conversion, image resizing, conversion to an array	Hybrid RviT (Pre-trained ResNet-50 + ViT)	Acc: 97 Prec: 93.75 Rec: 90.88 F1: 91.88	Hybrid ResNet50-ViT fusion improved local-global feature learning, but transformer integration increased optimization complexity and small-dataset dependency.
Asaduzzaman et al. (2025)	Resizing, augmentation, SMOTE	ALZENET: Ensemble of VGG16, Inception V3, ResNet50, and a custom CNN	Acc: 97.31 Prec: 97.37 Rec: 97.31 F1: 97.33	ALZENET improved multiclass classification through ensemble feature fusion, but SMOTE-dependent balancing and multi-backbone integration increased computational complexity and limited clinical generalizability.

### 3.5 Key findings

CNN-based approaches for Alzheimer's disease diagnosis have shown substantial progress through hybrid, ensemble, transfer learning, and attention-based architectures. Hybrid and ensemble frameworks such as ALZENET, ResViT, DenseNet-based systems, and optimization-assisted CNN models generally outperformed conventional standalone CNNs because they combined complementary feature representations and improved feature selection robustness (Sasithradevi et al., 2024; Fathi et al., 2024; Asaduzzaman et al., 2025). Multimodal MRI-PET frameworks further improved classification performance by integrating complementary structural and functional information, although these models introduced greater preprocessing and computational complexity (Tang et al., 2024). Preprocessing techniques including SMOTE, CLAHE, BADF filtering, augmentation, and segmentation improved image quality, reduced class imbalance, and enhanced model generalization (Nithya et al., 2024). Lightweight and depth-wise separable CNN architectures reduced computational complexity while maintaining competitive performance, indicating better scalability for practical deployment (Tripathy et al., 2024). Transformer-inspired and attention-based architectures improved early-stage disease characterization by capturing long-range spatial dependencies and subtle anatomical variations more effectively than conventional CNNs (Rahman et al., 2025). However, reported high accuracies should be interpreted cautiously because performance varies depending on datasets, preprocessing pipelines, validation strategies, and classification settings. Many studies relied heavily on benchmark datasets such as ADNI with limited external validation, raising concerns regarding overfitting, dataset bias, and real-world clinical generalizability. Overall, CNN-based models show strong potential for automated Alzheimer's disease diagnosis, but challenges related to explainability, computational complexity, standardized benchmarking, and clinical validation remain major barriers to routine clinical translation.

### 3.6 Limitations

Several challenges remain in CNN-based AD classification studies. Many models rely heavily on benchmark datasets such as ADNI and OASIS, potentially limiting generalizability across diverse clinical populations. Accurate differentiation of early disease stages, particularly MCI and mild dementia, remains difficult because of subtle structural brain changes and class imbalance. In addition, multimodal and hybrid CNN frameworks require extensive preprocessing and high computational resources, limiting practical clinical deployment. Limited external validation and reduced interpretability of complex deep learning architectures further restrict reliable clinical translation.

## 4 CNN in PD diagnosis and classification

### 4.1 Datasets

**Figure 4:**
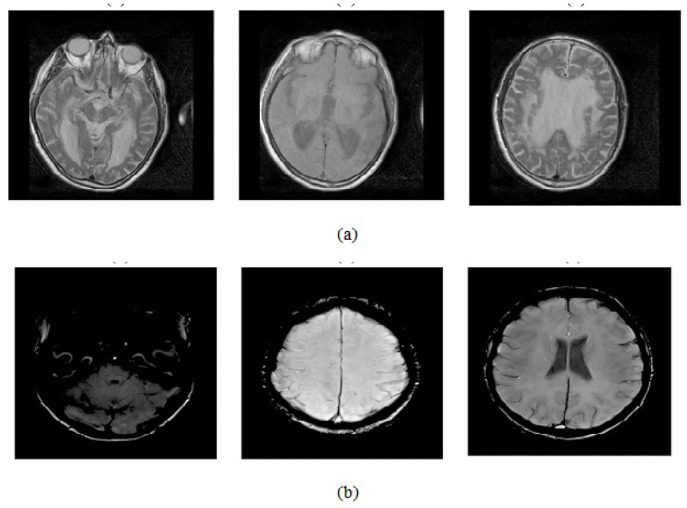
MRI images **(a)** PD patients, **(b)** normal people.

**Figure 5:**
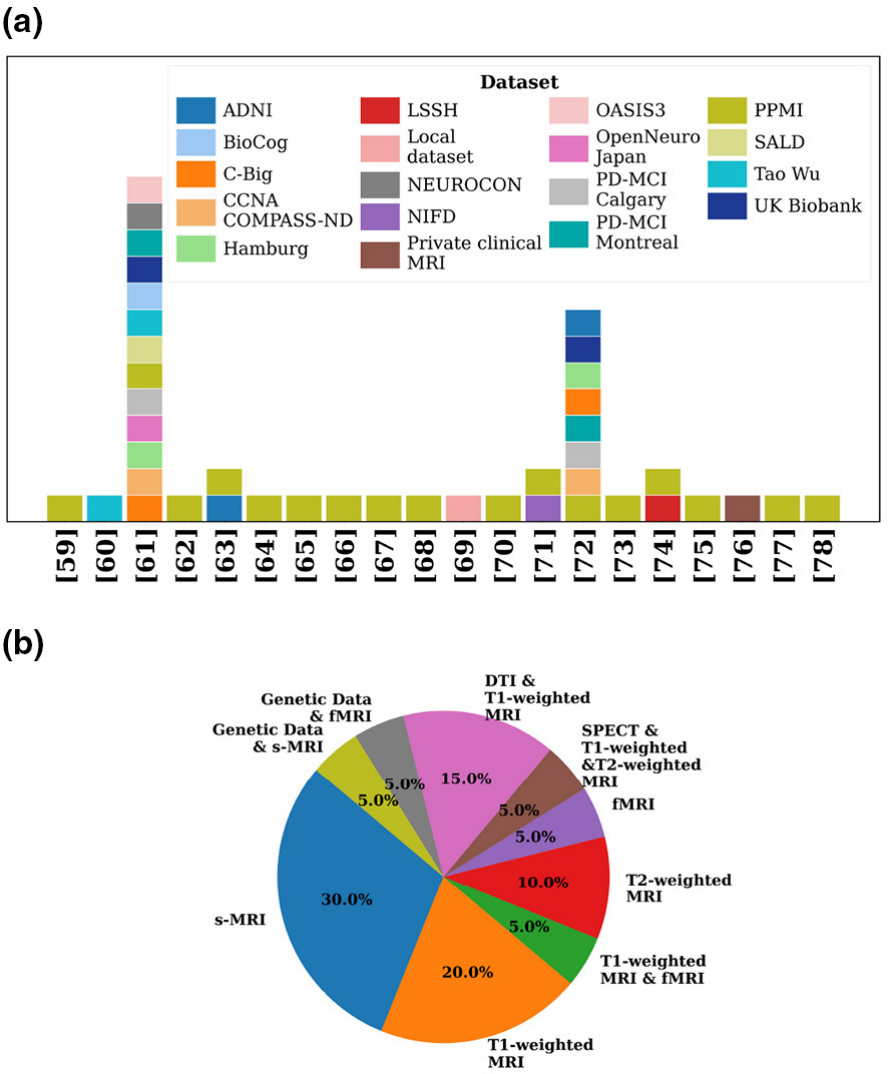
Dataset and imaging modality distribution in CNN-based Parkinson's disease studies: **(a)** dataset usage across reviewed studies, **(b)** image modality distribution in PD.

Parkinson's progression markers initiative (PPMI) is the most popular dataset for PD diagnosis. It contains neuroimaging, genetic and clinical data for PD and controls (Cui et al., 2023). Other available datasets for PD analysis include ADNI, UK Biobank, and Tao Wu (Islam et al., 2023; Camacho et al., 2023). studies have also used local data from institutions like Ningxia Medical University and Lalitha Super Specialty Hospital (LSSH). These datasets are unavailable for public use because of privacy reasons; thus, their reproducibility is restricted. The MRI samples of PD patients and normal people are shown in Figure 4. There is a significant proportion of multimodal applications towards PD diagnosis that integrate s-MRI, T1-weighted MRI, T2-weighted MRI, fMRI, SPECT, diffusion tensor imaging (DTI), and genetic information (Majhi et al., 2024; Karthigeyan and Rani, 2024). This enables deeper feature extraction, enhancing model performance in PD classification. The dataset usage across reviewed studies and Image modality distribution in PD are shown in Figures 5a, b. Table 3 summarizes the datasets, modalities, dataset sizes, classification categories, validation strategies, and dataset availability employed in the reviewed PD based studies.

**Table 3:** Summary of datasets, modalities, validation methods, and dataset availability employed in reviewed PD classification studies.

References	Dataset/ modality	Dataset size	Classes	Validation method	Dataset availability
Cui et al. (2023)	PPMI (MRI)	504 subjects (255 PD, 249 HC)	PD vs. HC (binary)	80:20 train-test split with fivefold cross-validation	https://www.ppmi-info.org
Islam et al. (2023)	Tao Wu dataset (T1 MRI + resting-state fMRI)	40 subjects (20 PD, 20 HC)	PD vs. HC (binary)	10-fold cross-validation with a separate 10% test set	https://fcp-indi.s3.amazonaws.com/data/Projects/INDI/umf_pd/taowu.tar.gz
Camacho et al. (2023)	Multi-dataset (T1-weighted MRI)	2,041 subjects (1024 PD, 1017 HC)	PD vs. HC (binary)	85:5:10 train-validation-test split with independent test-set validation	PPMI: https://www.ppmi-info.org/data C-BIG: https://www.mcgill.ca/neuro/open-science/c-big-repository NEUROCON/Tao Wu: http://fcon_1000.projects.nitrc.org/indi/retro/parkinsons.html OpenNeuro Japan: https://openneuro.org/datasets/ds000245/versions/00001 UK Biobank: https://pan.ukbb.broadinstitute.org Tao Wu Dataset: http://fcon_1000.projects.nitrc.org/indi/retro/parkinsons.html SALD: http://fcon_1000.projects.nitrc.org/indi/retro/sald.html
Majhi et al. (2024)	PPMI (T1/T2-weighted MRI and SPECT DaTscan)	66 subjects (33 PD, 33 HC); 29,166 images (9,070 MRI, 20,096 SPECT)	PD vs. HC (binary)	80:20 train-test split; training data further divided into 80:20 train-validation sets	https://www.ppmi-info.org
Karthigeyan and Rani (2024)	PPMI and ADNI (rfMRI + Gene data)	104 subjects (55 PD from PPMI; 35 HC from ADNI and 14 HC from PPMI)	PD vs. HC (binary)	6-fold cross-validation	PPMI: https://www.ppmi-info.org ADNI: https://adni.loni.usc.edu
Palakayala and Kuppusamy (2024a)	PPMI (T1-weighted MRI)	500 MRI scans (250 PD, 250 HC); augmented to 2,500 images	PD vs. HC (binary)	80:20 train-validation/test split with 10-fold cross-validation	https://www.ppmi-info.org/data
Praneeth et al. (2023)	PPMI (T1-weighted MRI and diffusion MRI (dMRI))	591 subjects (412 PD, 179 HC)	PD vs. HC (binary)	Cross-validation evaluation	https://www.ppmi-info.org
Erdaş and Sümer (2023)	PPMI (T1-weighted MRI)	1,130 MRI scans (871 PD, 259 HC)	PD vs. HC; PD severity prediction	10-fold cross-validation	https://www.ppmi-info.org/access-data-specimens/download-data
Kumar et al. (2024)	PPMI (T1-weighted MRI and diffusion MRI (dMRI))	591 subjects (412 PD, 179 HC)	PD vs. HC (binary)	80% training, 20% testing	https://www.ppmi-info.org
Acikgoz et al. (2024)	PPMI (T2-weighted axial MRI)	480 subjects (320 PD, 160 HC)	PD/HC	70:15:15 train-validation-test split	https://www.ppmi-info.org
Welton et al. (2024)	3T Midbrain MRI (QSM, SMWI and NMS-MRI)	189 subjects (82 PD, 107 HC)	PD/HC	Independent external validation	Available on reasonable request
Sailaja and VenuGopal (2023)	PPMI (Brain MRI scans)	831 images	PD/HC	70:30 train-test split (582 train, 249 test)	Available on reasonable request
Patil and Ford (2024)	PPMI + NIFD (rs-fMRI)	398 subjects (183 PPMI: 164 PD, 19 HC; 215 NIFD HC)	PD/HC	70% training, 15% validation, 15% testing	PPMI: https://www.ppmi-info.org NIFD: https://memory.ucsf.edu/research-trials/research/allftd
Camacho et al. (2024)	Multi-dataset (T1-weighted MRI + DTI)	1,264 subjects (611 PD, 653 HC)	PD/HC	85% training, 5% validation, 10% testing	PPMI: https://www.ppmi-info.org/data C-BIG: https://www.mcgill.ca/neuro/open-science/c-big-repository ADNI: https://adni.loni.usc.edu UK Biobank: https://pan.ukbb.broadinstitute.org
Kumari et al. (2024)	PPMI (T1-weighted MRI)	7,240 images (3,620 PD, 3,620 HC)	PD/HC	80% training, 20% testing	https://www.ppmi-info.org/access-data-specimens/download-data
Palakayala and Kuppusamy (2024b)	PPMI + LSSH (T1-weighted MRI)	100 images (60 PD, 40 HC)	PD/HC	70% training, 20% validation, 10% testing	PPMI: https://www.ppmi-info.org/data; LSSH: private clinical dataset
Mahendran and Visalakshi (2024)	PPMI (DaTscan SPECT)	645 images (432 PD, 213 non-PD)	PD/non-PD	80% training, 20% testing	https://www.ppmi-info.org
Li et al. (2024)	Private clinical MRI dataset (3T MRI)	104 subjects, 582 images (54 PD, 50 HC)	PD/HC	80% training, 20% testing	Available on reasonable request
Li et al. (2025)	PPMI (sMRI + SNP genetic data)	450 subjects (308 PD, 142 HC)	PD/HC	5-fold cross-validation	https://www.ppmi-info.org
Ding et al. (2025)	PPMI (T1-weighted MRI + DTI)	544 images (308 MRI, 236 DTI)	PD/HC	70% training, 30% testing	http://www.ppmi-info.org/access-data-specimens/download-data

### 4.2 Preprocessing and data augmentation

Preprocessing for MRI- and fMRI-based PD diagnosis primarily involved DICOM-to-NIfTI conversion, skull stripping, MNI alignment or atlas registration, normalization, filtering, smoothing, and denoising (Islam et al., 2023; Palakayala and Kuppusamy, 2024a). Several studies additionally employed augmentation and enhancement strategies such as rotation, flipping, contrast enhancement, and adaptive filtering to improve robustness and reduce overfitting (Cui et al., 2023; Kumar et al., 2024).

### 4.3 Segmentation

For PD, Dense-UNet was implemented to address the resolution loss typically seen in traditional U-Net architectures due to successive downsampling (Praneeth et al., 2023). Dense-UNet enhances segmentation accuracy by using dense connections that facilitate greater information flow and reduce redundancy in feature maps. The architecture is effective in ensuring spatial resolution and enhancing the efficiency and effectiveness of the model in brain MRIs segmentation tasks. To further enhance segmentation accuracy, the hybrid AttAggUNet model leverages the benefits of both the attention mechanism and the aggregate feature fusion strategy, which combines the two models of AttUNet and AggUNet (Palakayala and Kuppusamy, 2024a).

### 4.4 CNN models

A diverse array of DL models has been developed for PD diagnosis, ranging from classic CNN to advanced hybrid and attention-based architectures. Early models used 2D and 3D CNNs, frequently based on architectures such as AlexNet (Erdaş and Sümer, 2023). More recent methods incorporate attention mechanisms and multi-branch processing, such as adaptive attention feature processing module-DL (AAFP-DL) and attention-based deep-stack convolutional hunter-prey network (ADeepSCHN) or hybridized networks such as LeNet-5 with U-Net (AttentionLUNet) (Kumar et al., 2024). In addition, models such as feature extraction-decorrelated CNN (FE-DcCNN) overcome the issue of scanner variability, and models like Res-DenseNet or attention and multi-level enhancement fusion network (AMEF-Net) add dense blocks, attention modules, and deformable convolutions to enhance feature extraction (Acikgoz et al., 2024). Another widely adopted approach are ensemble methods that fuse architectures such as VGG16, ResNet and EfficientNet to enhance classification robustness. Lightweight models and domain-specific solutions like Heuron IPD and Parkinson's integrative diagnostic gated network (PIDGN) further demonstrate the diversity and innovation in PD model design (Welton et al., 2024). The transition from traditional CNNs to hybrid, attention-based, and ensemble models highlights the difficulty in identifying subtle patterns from Parkinson's disease imaging and the requirement for more discriminative feature representations. Table 4 summarizes the different models used and their performance for PD diagnosis across the studies.

**Table 4:** Summary of CNN-based DL studies for PD diagnosis using MRI data.

References	Data preparation	Model	Performance metrics	Architectural trade-offs and limitations
Cui et al. (2023)	Normalize, denoise, slice, augmentation	AAFP-DL: deep CNN with attention mechanisms (MuSENet) and multi-branch processing	Acc: 90; Rec: 92; F1: 91	Multi-scale attention and adaptive feature fusion improved lesion-aware feature representation, but heavy augmentation and branch-wise attention weighting increased preprocessing dependency and optimization complexity.
Islam et al. (2023)	Alignment to MNI152, skull stripping, motion correction, smoothing, filtering	3D CNN with convolution, pooling, dropout	Acc: 86.07; Rec: 87.4	ICA-based RSN extraction improved network-level feature learning, but small single-center data and preprocessing-intensive pipelines limited generalizability.
Camacho et al. (2023)	Brain extraction, resampling, bias correction, registration, Log-Jacobian maps	3D CNN with batch normalization and ReLU	Acc: 79.3; Prec: 80.2; Rec: 77.7	3D Jacobian-based CNN improved explainable PD classification across multicenter MRI data, but registration-dependent preprocessing and moderate diagnostic accuracy may limit clinical reliability.
Majhi et al. (2024)	Image conversion, cropping, skull stripping, normalization	GWO-VGG16 + InceptionV3	Acc: 99.92; Prec: 99.9; Rec: 99.81; F1: 100	Hybrid GWO-VGG16 + InceptionV3 improved PD classification, but preprocessing-dependent training and high architectural complexity may limit real-world generalizability.
Karthigeyan and Rani (2024)	Normalization, scaling	ABCNN + stacked CNN-LSTM	Acc: 99.4; Prec: 99; Rec: 99.5; F1: 99	Multimodal CNN-LSTM fusion improved complementary rfMRI-gene feature learning, but stacked attention-based architecture and modality dependency increased optimization complexity.
Palakayala and Kuppusamy (2024a)	NIfTI conversion, skull stripping, normalization, filtering, augmentation	AttentionLUNet (LeNet + U-Net + attention)	Acc: 99.58; Prec: 99.60; Rec: 99.60; F1: 99.60	AttentionLUNet improved PD classification through hybrid LeNet-U-Net feature learning, but preprocessing-intensive training and architectural complexity may limit generalizability.
Praneeth et al. (2023)	Min-max normalization, median filtering	Dense-Unet + residual CNN	Acc: 98.87; Prec: 97.02; Rec: 96.87	Dense-UNet and DRCNN improved PD feature learning, but dense connectivity and optimization-based training increased model dependency.
Erdaş and Sümer (2023)	FLIRT registration, BET skull stripping	2D CNN + 3D CNN	Acc: 96.2; Prec: 94.07; Rec: 95.36; F1: 94.52	Automated FLIRT-BET preprocessing and combined 2D/3D CNN learning improved PD analysis, but MRI downsampling and simplified 3D CNN design may limit fine-grained feature representation.
Kumar et al. (2024)	Adaptive frost filtering	Deep stacked CNN with attention	Acc: 99.61; Prec: 98.96; Rec: 98.72; F1: 99.41	Attention-based deep stacked CNN improved PD classification, but optimization-dependent feature selection may limit scalability.
Acikgoz et al. (2024)	Augmentation	Res-DenseNet with attention	Acc: 94.4; Prec: 91.67; Rec: 91.67; F1: 91.67	Residual DenseNet with SE-ResNeXt improved multiscale feature learning, but dense attention connectivity increased parameter overhead.
Welton et al. (2024)	FSL preprocessing, manual segmentation	CNN-based analysis and segmentation	Heuron IPD, Heuron NI volume Acc: 90.48, 84.57; Rec: 100, 83.95	Heuron IPD improved PD classification, but artifact-sensitive SMWI processing and proprietary CNN segmentation limited robustness.
Sailaja and VenuGopal (2023)	Not specified	ResNet50v2	Acc/Prec/Rec: 100	Modified ResNet50V2 improved PD classification through residual feature learning, but dense skip-connected architecture and small MRI datasets may limit robustness and generalizability.
Patil and Ford (2024)	Brain extraction, motion correction, smoothing, filtering	Dual CNN for feature extraction and classification	DcCNN, FE-DcCNN Acc: 80.47, 77.80; Prec: 80.43, 75.03; Rec: 79.63, 80.53; F1: 79.57, 77.13	Decorrelated CNNs improved PD recognition by reducing class and scanner bias in rs-fMRI data, but multistage decorrelation learning increased architectural and training complexity.
Camacho et al. (2024)	DTI processing, MRI mapping, atlas alignment	Lightweight 3D CNN	Acc: 80.8; Rec: 79.1	Multimodal CNN improved PD classification, but multimodal fusion may introduce scanner-specific feature bias.
Kumari et al. (2024)	Resizing, normalization	DenseNet201 (modified)	Acc: 99.2; Prec: 99.3; Rec: 99.1; F1: 99.2	DenseNet201 improved PD detection, but dense connectivity and single-cohort training may limit generalizability.
Palakayala and Kuppusamy (2024b)	DICOM to NIfTI, skull stripping, normalization, filtering	Ensemble CNNs (EfficientNet, ResNet, MobileNet)	Acc: 99; Prec: 99.3; Rec: 98.6; F1: 98.99	Ensemble DCNN improved PD classification, but multimodel integration increased model dependency on augmented MRI data.
Mahendran and Visalakshi (2024)	Resizing, normalization, artifact removal	Ensemble CNNs with fusion ranking	Acc: 98.92; Prec: 98.84; Rec: 98.84; F1: 98.84	Ensemble CNNs with fuzzy fusion improved PD classification, but multimodel fusion increased computational and architectural complexity.
Li et al. (2024)	Manual labeling, data shuffling	YOLOv5-based CNN with attention	Prec: 96.1; Rec: 97.4	Attention-enhanced YOLOv5 improved PD MRI detection, but increased architectural complexity and depended on limited single-center data.
Li et al. (2025)	NIfTI conversion, skull stripping, MNI alignment	ResNet18 + PIDGN	Acc: 85.8; Prec: 90.1; Rec: 90; F1: 90.1	PIDGN improved PD prediction through multimodal fusion, but relied on extensive preprocessing and high-dimensional multimodal integration.
Ding et al. (2025)	Filtering, skull stripping, MNI registration, DTI processing	AMEF-Net (EfficientNet + attention)	MRI, DTI Acc: 98.867, 99.602; Prec: 99.830, 99.930; Rec: 99.182, 99.463	AMEF-Net improved PD classification through multilevel attention fusion, but depended on extensive preprocessing and multimodal MRI/DTI integration.

### 4.5 Key findings

CNN-based approaches for Parkinson's disease diagnosis have evolved from conventional CNNs toward hybrid, multimodal, attention-based, and optimization-assisted architectures. Attention mechanisms, transfer learning, and modified deep networks improved feature extraction, convergence stability, and classification robustness compared with standalone CNN models by enhancing subtle neurodegenerative pattern representation and reducing feature redundancy (Cui et al., 2023; Sailaja and VenuGopal, 2023; Palakayala and Kuppusamy, 2024a). Hybrid and optimization-assisted frameworks combining DRCNN, GWO, VGG16, and InceptionV3 also demonstrated strong classification performance (Praneeth et al., 2023; Majhi et al., 2024). Multimodal frameworks integrating MRI, fMRI, DTI, SPECT, and genetic information improved diagnostic capability by combining complementary structural, functional, and genomic biomarkers, although these approaches increased preprocessing complexity and data harmonization requirements (Islam et al., 2023; Karthigeyan and Rani, 2024; Li et al., 2025). Explainable AI approaches using SHAP analysis, Grad-CAM, Jacobian mapping, and attention mechanisms further improved interpretability by identifying clinically relevant brain regions and biomarkers associated with PD (Camacho et al., 2023; Li et al., 2025). However, reported performance metrics should be interpreted cautiously because several studies achieved exceptionally high accuracies using limited or single-source datasets under controlled experimental settings (Sailaja and VenuGopal, 2023; Majhi et al., 2024). Many studies relied heavily on benchmark datasets such as PPMI with limited external validation, raising concerns regarding dataset bias, overfitting, and reduced real-world clinical generalizability. Although multimodal and optimization-assisted frameworks improved classification performance, they often introduced higher computational complexity and reduced deployment feasibility. Overall, reliable clinical translation will require improved multicenter validation, explainable biomarker identification, and computationally efficient frameworks suitable for real-world neurological practice.

### 4.6 Limitations

PD classification studies remain challenged by small cohorts, scanner variability, heterogeneous acquisition protocols, and limited external validation. Many models rely on binary PD-HC classification, which may inadequately capture disease progression or distinguish PD from related neurodegenerative disorders. In addition, subtle structural brain changes and multimodal harmonization difficulties across MRI, fMRI, and DTI data can affect robustness and reproducibility. Although advanced hybrid and attention-based frameworks improve classification performance, their computational demands and limited interpretability continue to restrict practical clinical translation.

## 5 CNN in brain tumor diagnosis and classification

### 5.1 Datasets

**Figure 6:**
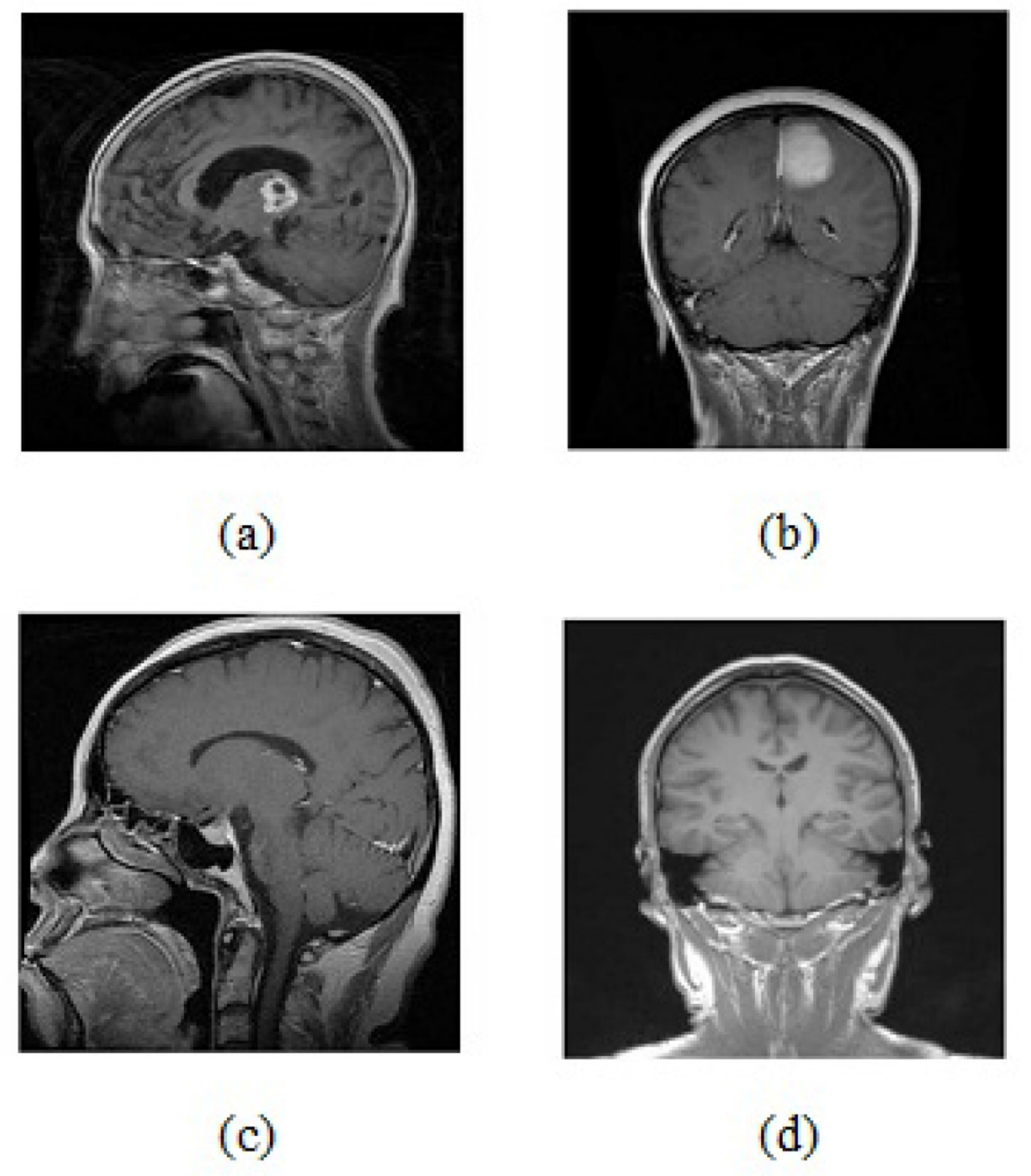
MRI images of BT: **(a)** glioma, **(b)** meningioma, **(c)** pituitary, **(d)** no tumor.

**Figure 7:**
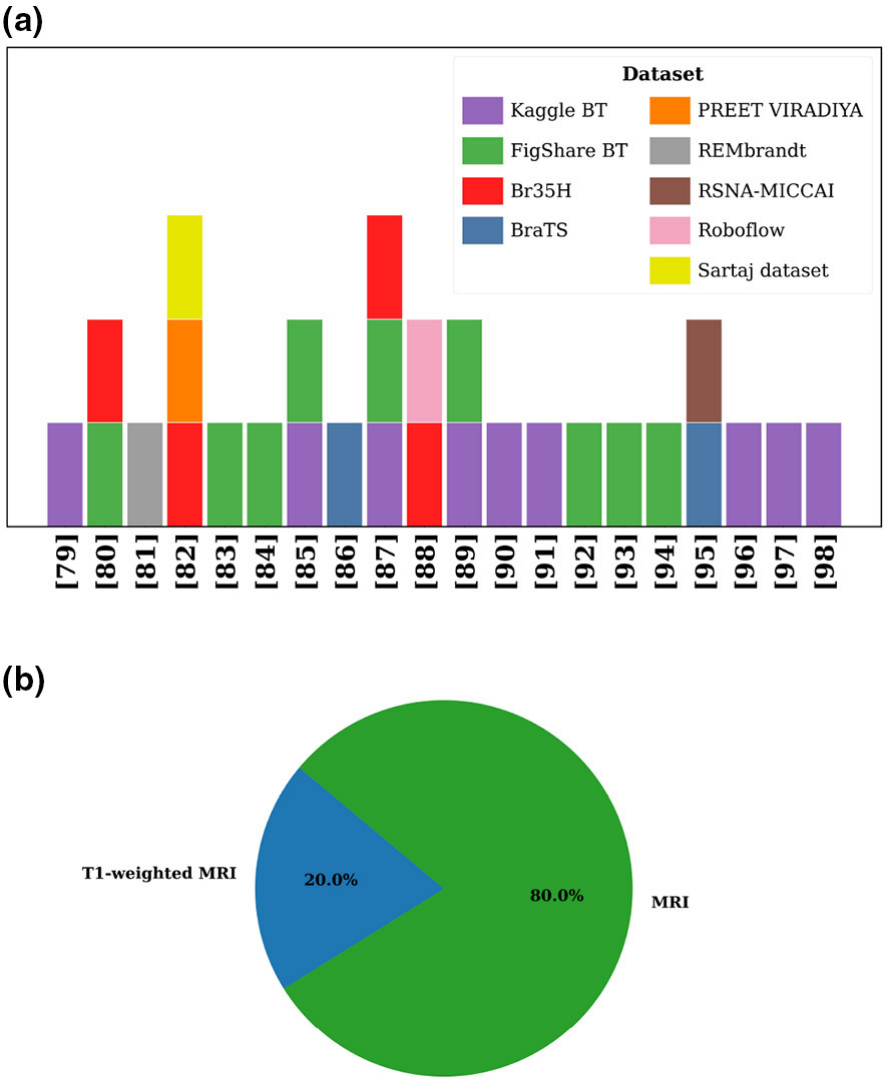
Dataset and imaging modality distribution in CNN-based brain tumor studies: **(a)** dataset usage across reviewed studies, **(b)** image modality distribution in brain tumor.

Kaggle BT and FigShare are the most commonly employed datasets for brain tumor diagnosis and classification, followed by Br35H and BraTS. The Kaggle BT dataset consists of 7,023 brain MRI scans classified into four classes, namely glioma (1,621), healthy (2,000), meningioma (1,645), and pituitary tumor (1,757) (Khaliki and Başarslan, 2024). FigShare dataset consists of 3,064 images categorized into meningioma (708), glioma (1,426), and pituitary tumors (930) (Bhardwaj et al., 2024). All the studies employ MRI as a single modality for the diagnosis and classification. The samples of MRI images for different classes of tumor are shown in Figure 6. This proves that MRI remains the gold standard for brain tumor detection. Figures 7a, b depicts the dataset usage across reviewed studies and Image modality distribution in brain tumors. Table 5 summarizes the datasets, modalities, dataset sizes, classification categories, validation strategies, and dataset availability employed in the reviewed brain tumor based studies.

**Table 5:** Summary of datasets, modalities, validation methods, and dataset availability employed in reviewed brain tumor-based studies.

References	Dataset/ modality	Dataset size	Classes	Validation method	Dataset availability
Khaliki and Başarslan (2024)	Kaggle Brain Tumor MRI dataset (MRI)	2,870 MRI images (926 glioma, 937 meningioma, 440 no-tumor, 901 pituitary)	Glioma/meningioma/ no-tumor/pituitary (4-class)	75:15:10 train-validation-test split	https://doi.org/10.34740/KAGGLE/DSV/1183165
Bhardwaj et al. (2024)	BR35H Brain Tumor Detection 2020 and Figshare dataset (MRI)	3,000 binary MRI images (1,500 tumor, 1,500 non-tumor); 2,400 multiclass MRI images (800 glioma, 800 meningioma, 800 pituitary)	Tumor/non-tumor (binary); glioma/meningioma/pituitary (3-class)	Validation split of 0.2 or 0.3 during training	Not specified
Mithun and Jawhar (2024)	REMBRANDT dataset (MRI)	3,200 MRI images (670 glioma, 705 meningioma, 815 pituitary, 1,010 no-tumor)	Glioma/meningioma/ no-tumor/pituitary (4-class)	80:20 train-test split with 10-fold cross-validation	Not specified
Islam et al. (2024)	Br35H, Sartaj, and PREET VIRADIYA Brain Tumor MRI datasets	10,470 MRI images (3,000 Br35H, 1,311 Sartaj, 4,600 PREET VIRADIYA)	Brain tumor/healthy (binary)	75:25 train-test split	Publicly available Kaggle datasets
Ilani et al. (2025)	Figshare dataset (T1-weighted contrast-enhanced MRI)	3,064 MRI images (1,426 glioma, 708 meningioma, 930 pituitary)	Glioma/meningioma/pituitary (3-class)	80:20 train-test split with 5-fold stratified cross-validation	Publicly available
Alamri et al. (2025)	Figshare Brain Tumor MRI dataset	3,064 MRI images (1,426 glioma, 708 meningioma, 930 pituitary)	Glioma/meningioma/pituitary (3-class)	Training and testing evaluation	https://www.kaggle.com/datasets/ashkhagan/figshare-brain-tumor-dataset
Dutta et al. (2025)	Figshare and Kaggle datasets (MRI)	Not specified	Glioma/meningioma/pituitary (3-class)	Not specified	Publicly available
Rahman et al. (2024)	BraTS 2020 dataset (MRI)	94,296 MRI images (24,628 LGG, 69,668 HGG)	LGG / HGG (binary)	70:10:20 train-validation-test split	Available on reasonable request
Zahoor et al. (2024)	Kaggle, Br35H, and Figshare datasets (MRI)	7,872 MRI images (2,352 glioma, 1,645 meningioma, 1,831 pituitary, 2,044 normal)	Glioma/meningioma/pituitary/ normal (4-class)	80:20 train-test split with 10% validation using holdout cross-validation	Publicly available
Huang et al. (2025)	Br35H and Roboflow brain tumor MRI datasets (MRI)	877 MRI images	Brain tumor/healthy	Br35H: 561 train, 160 test, 80 validation; Roboflow: 60 train, nine test, seven validation	Publicly available
Su et al. (2025)	Kaggle MRI dataset and Figshare dataset	7,023 MRI images (1,621 glioma, 1,645 meningioma, 1,757 pituitary, 2,000 no-tumor); 3,064 MRI images (1,462 glioma, 708 meningioma, 930 pituitary)	Glioma/meningioma/pituitary/ no-tumor (4-class); Glioma/meningioma/pituitary (3-class)	Train-test split (Kaggle); 80:20 train-test split (Figshare)	https://www.kaggle.com/datasets/masoudnickparvar/brain-tumor-mri-dataset; https://figshare.com
Ragab et al. (2024)	Kaggle Brain MRI dataset (MRI)	251 MRI images (153 tumor, 98 no-tumor)	Tumor/no-tumor (binary)	80:20 and 70:30 train-test split	Available on reasonable request
Saboor et al. (2024)	Kaggle brain MRI dataset (MRI)	1,311 MRI images (300 glioma, 306 meningioma, 300 pituitary, 405 normal)	Glioma/meningioma/pituitary/no-tumor (4-class)	70:30 train-test split using hold-out cross-validation	https://www.kaggle.com/datasets/shreyag1103/brain-mri-scans-for-brain-tumor
Agarwal et al. (2024)	Figshare CE-MRI dataset (MRI)	3,461 T1-CE MRI images from 233 patients (780 benign, 2,681 malignant)	Benign/malignant (2-class)	Training set: 3,064 images; test set: 397 images	Available on request
Xu and Mohammadi (2024)	Figshare dataset (MRI)	3,064 MRI images from 233 patients (1,426 glioma, 930 pituitary, 708 meningioma)	Glioma/pituitary/meningioma	80:20 train-test split	https://figshare.com/articles/dataset/brain_tumor_dataset/1512427/5
Aziz et al. (2024)	Figshare dataset (MRI)	3,064 MRI images from 233 patients (1,426 glioma, 930 pituitary, 708 meningioma)	Glioma/pituitary/meningioma	80:20 train-test split	https://figshare.com/articles/dataset/brain_tumor_dataset/1512427/5
Raza and Iqbal (2025)	BraTS and RSNA-MICCAI Brain Tumor datasets (MRI)	1,540 MRI images (1,140 BraTS, 400 RSNA-MICCAI), augmented with 1,131 additional images	Tumor/healthy	7:2:1 train-test-validation split	https://www.med.upenn.edu/cbica/brats2020/data.html
Disci et al. (2025)	Kaggle brain tumor MRI Dataset (MRI)	7,023 MRI images (1,621 glioma, 1,645 meningioma, 1,757 pituitary, 2,000 no-tumor)	Glioma/meningioma/pituitary/no-tumor (4-class)	Predefined train-test split	https://www.kaggle.com/datasets/masoudnickparvar/brain-tumor-mri-dataset
Mao et al. (2025)	Kaggle brain tumor MRI Dataset (MRI)	7,023 MRI images (1,621 glioma, 1,645 meningioma, 1,757 pituitary, 2,000 no-tumor)	Glioma/meningioma/pituitary/no-tumor (4-class)	Predefined train-test split with 10-fold cross-validation	https://doi.org/10.34740/KAGGLE/DSV/2645886
Batool and Byun (2025)	Kaggle brain tumor MRI Dataset (MRI)	3,264 T1-weighted contrast-enhanced MRI images (926 glioma, 937 meningioma, 500 pituitary, 901 no-tumor)	Glioma/meningioma/pituitary/no-tumor (4-class)	10-fold cross-validation	Not specified

### 5.2 Preprocessing and data augmentation

Preprocessing for brain tumor MRI analysis mainly involved grayscale conversion, ROI extraction or cropping, resizing, normalization, filtering, and noise reduction (Mithun and Jawhar, 2024; Disci et al., 2025). Several studies additionally employed enhancement and augmentation strategies such as contrast enhancement, histogram equalization, rotation, flipping, scaling, and translation to improve tumor visualization, robustness, and classification performance (Agarwal et al., 2024; Islam et al., 2024).

### 5.3 Segmentation

Segmentation of brain tumors has significantly benefited from advanced deep learning models, particularly U-Net and its variants. U-Net, known for its encoder-decoder architecture, efficiently processes biomedical images and has been adapted for 3D MRI segmentation (Ilani et al., 2025). The En-DeNet model extends U-Net by incorporating EfficientNet-b3 as the encoder to improve feature extraction and generalization (Mithun and Jawhar, 2024). The decoder uses multiple convolutional blocks to derive tumor properties, with training optimized using a combination of dice loss and binary cross-entropy, later enhanced with Hausdorff distance for contour accuracy. Further refinement is provided by incorporating edge and region-based segmentation through QE edge detection and NAdam optimized U-Net (Alamri et al., 2025). Furthermore, the NSAS-Net model is a nested self-attentive network for processing images at multiple scales to improve the capability of discriminating tumor size and shape (Dutta et al., 2025). The self-attention mechanism creates attention maps to boost the tumor boundary detection and segmentation accuracy and utilize the context information.

### 5.4 CNN models

CNNs are extensively used in brain tumor diagnosis and classification, with various model architectures proving effective for feature extraction from neuroimaging data. The most common models are the traditional 3-layer CNNs with pooling, as well as more sophisticated models such as InceptionV3, VGG16, VGG19, and EfficientNetB4 (Khaliki and Başarslan, 2024). Other notable models include SE-ResNet50, stacked autoencoders (SAE), and lightweight CNNs such as GliomaCNN, which employ GELU activation along with convolutional and fully connected layers (Rahman et al., 2024). CNN-LSTM and CNNRes-BRNet, combining spatial block and residual block, have also shown great performance in hybrid architectures (Zahoor et al., 2024). Additionally, models such as YOLO NAS, EfficientDet, and YOLO-TumorNet, which focus on multi-scale feature extraction, are increasingly used for more complex classification tasks (Huang et al., 2025). Specialized models such as DeepNeuroXpert, combining NSAS Net and AI2CF, and ParMamba, featuring convolutional attention and channel enhancement, further highlight the versatility of CNNs in brain tumor classification (Su et al., 2025). As a result, the shift from classical CNNs to hybrid, attention-based, and multi-scale CNNs has emerged, driven by the desire to accommodate the complexity of tumor heterogeneity, precise localization of tumor patterns, and stronger robustness to classification across various tumor types. Table 6 summarizes the various CNN-based architectures for diagnosing and classifying brain tumor in neuroimaging studies and their performance metrics.

**Table 6:** Summary of CNN-based DL studies for brain tumor diagnosis using MRI data.

References	Data preparation	Model	Performance metrics	Architectural trade-offs and limitations
Khaliki and Başarslan (2024)	Not specified	CNN variants (InceptionV3, VGG16, VGG19, EfficientNetB4)	VGG16, CNN Acc: 98, 91; Prec: 98, 91; Rec: 98, 92; F1: 97, 90	Transfer learning CNNs improved brain tumor classification, but deep pretrained architectures and low-resolution MRI inputs may limit feature generalization.
Bhardwaj et al. (2024)	Resizing, shuffling, normalization, augmentation	CNN for binary and multiclass classification	CNN1, CNN2 Acc: 98.56, 92.36; Prec: 99, 92; Rec: 99, 93; F1: 99, 94	Lightweight CNN reduced architectural complexity, but limited hierarchical feature learning may affect generalization.
Mithun and Jawhar (2024)	Grayscale conversion, resizing, ROI selection, noise removal	YOLO-NAS and EfficientDet with attention	Acc: 99.7; Prec: 98.2; Rec: 98.5; F1: 99.2	Hybrid YOLO-NAS and En-DeNet architecture increased multistage model dependency, but may limit scalability.
Islam et al. (2024)	Grayscale conversion, resizing, feature scaling, augmentation	2D CNN and hybrid CNN-LSTM model	Acc: 98.82; Prec: 99.73; Rec: 99.03; F1: 99	Hybrid CNN-LSTM architecture increased multistage feature integration and temporal dependency, but may reduce cross-dataset generalizability.
Ilani et al. (2025)	Resizing, cropping, shuffling, augmentation	U-Net, CNN, transfer learning models	Acc: 98.56; Prec: 99; Rec: 99; F1: 99	U-Net and transfer learning architectures improved multiscale feature extraction, but deep pretrained backbones increased parameter dependency and overfitting risk.
Alamri et al. (2025)	Wiener filtering, gamma correction	UNet with GCRNN classifier	Acc: 98; Prec: 96.7; Rec: 97.1; F1: 96.89	Multiscale feature fusion increased architectural dependency and optimization overhead.
Dutta et al. (2025)	Noise reduction, normalization, skull stripping, augmentation	DeepNeuroXpert architecture	Acc: 99.4; Prec: 99; Rec: 99; F1: 99	Nested self-attention and capsule fusion architecture improved hierarchical feature extraction, but increased multistage training and optimization complexity.
Rahman et al. (2024)	3D to 2D conversion, cropping, filtering	Lightweight CNN (GliomaCNN)	Acc: 99.16; Prec: 99.05; Rec: 99.15; F1: 99.14	Lightweight GliomaCNN architecture reduced computational complexity, but shallow feature extraction and boosting dependency may limit generalizability.
Zahoor et al. (2024)	Resizing, augmentation	CNNRes-BRNet with residual blocks	Acc: 98.22; Prec: 98.22; Rec: 98.11; F1: 96.41	Res-BRNet combined spatial and residual blocks to improve multiscale feature learning, but increased architectural complexity and training dependency.
Huang et al. (2025)	Not specified	YOLO-TumorNet (YOLO + InceptionNeXt + Bi-FPN)	Roboflow, Br35H Acc: 97.71, 99.50; Prec: 98.66, 99.47; F1: 97.62, 99.44	Multi-scale feature fusion and attention integration improved tumor localization and boundary representation, but increased architectural complexity and computational dependency.
Su et al. (2025)	Resizing, normalization, augmentation	ParMamba architecture	Kaggle, FigShare Acc: 99.62, 99.35; Prec: 99.58, 99.19; Rec: 99.59, 99.35; F1: 99.59, 99.27	Parallel CNN-Mamba integration improved local-global feature learning, but increased architectural complexity and feature-fusion dependency.
Ragab et al. (2024)	Median filtering	SE-ResNet50 and stacked autoencoder	Acc: 98.78; Prec: 98.12; Rec: 98.78; F1: 98.43	BTR-EODLA combined SE-ResNet50 feature extraction, EO-based hyperparameter tuning, and SAE classification, but increased multistage optimization dependency and architectural complexity.
Saboor et al. (2024)	Resizing, augmentation	CNN with GRU and attention	Acc: 99.32; Prec: 100; Rec: 99.12; F1: 99.01	Attention-guided GRU architecture improved sequential feature learning and tumor-region focus, but increased training complexity, memory consumption, and optimization dependency.
Agarwal et al. (2024)	Thresholding, histogram equalization, augmentation	InceptionV3 CNN	Acc: 98.89; Rec: 97.17	The two-stage contrast-enhancement and transfer-learning framework improved tumor feature extraction and classification, but increased multistage processing dependency and computational complexity.
Xu and Mohammadi (2024)	Contrast enhancement, normalization, augmentation	MobileNetV2	Acc: 97.32; Prec: 97.68; Rec: 80.12; F1: 86.22	Lightweight feature extraction and metaheuristic optimization improved computational efficiency and convergence, but increased optimization dependency and training complexity.
Aziz et al. (2024)	Normalization, augmentation	DenseNet pretrained model	Acc: 97.1; Prec: 97; Rec: 97; F1: 97	Dense connectivity with transfer-learning fine-tuning improved feature reuse and model generalization, but increased training dependency on regularization and hyperparameter tuning.
Raza and Iqbal (2025)	Augmentation, annotation	Lightweight-CancerNet	Acc: 98; Prec: 98; Rec: 100; F1: 94	Lightweight feature extraction and anchor-free detection improved real-time efficiency, but reduced feature-representation capacity and generalization robustness.
Disci et al. (2025)	Filtering, thresholding, ROI cropping, resizing, augmentation	CNN models (Xception, MobileNetV2, ResNet, etc.)	Acc: 98.73; Prec: 95.5; Rec: 95.27; F1: 95.29	Transfer-learning integration with multiple deep CNN backbones improved hierarchical feature extraction and classification accuracy, but increased model-selection dependency and overfitting sensitivity.
Mao et al. (2025)	Cropping, resizing, normalization, Gaussian blur, augmentation	Dilated SE-DenseNet	Acc: 96.2; Prec: 96.5; Rec: 96.5; F1: 96.5	Dilated convolutions and SE attention improved contextual feature learning, but increased dilation-rate dependency and optimization complexity.
Batool and Byun (2025)	Filtering, ROI cropping, resizing, augmentation	Lightweight CNN with parallel paths	Acc: 96.03; Prec: 96; Rec: 92.02; F1: 96.02	Multi-path feature extraction with varying kernel sizes improved multiscale representation learning and classification performance, but increased feature-fusion dependency and architectural complexity.

### 5.5 Key findings

CNN-based brain tumor classification approaches have evolved from conventional CNNs toward transfer learning, ensemble, lightweight, attention-based, and hybrid architectures. Transfer learning models such as VGG16, InceptionV3, EfficientNet, and DenseNet generally outperformed simpler CNN architectures by improving feature extraction and multiclass classification performance (Khaliki and Başarslan, 2024; Ragab et al., 2024). Hybrid and ensemble frameworks integrating CNN-LSTM, attention mechanisms, GRU modules, and optimization algorithms further improved robustness and feature representation (Islam et al., 2024; Saboor et al., 2024; Dutta et al., 2025). Lightweight models such as GliomaCNN and CancerNet achieved competitive performance with lower computational complexity, indicating better scalability for practical deployment (Rahman et al., 2024; Raza and Iqbal, 2025). Advanced architectures including YOLO-based frameworks and ParMamba improved multi-scale feature extraction, contextual learning, and small tumor detection (Huang et al., 2025; Su et al., 2025). Explainable AI methods such as SHAP and Grad-CAM++ improved interpretability by identifying clinically relevant tumor regions (Rahman et al., 2024). In addition, preprocessing techniques including augmentation, denoising, segmentation, and contrast enhancement enhanced image quality and classification robustness (Agarwal et al., 2024). However, reported performance metrics should be interpreted cautiously because several studies achieved exceptionally high accuracies using limited benchmark datasets and controlled experimental settings (Rahman et al., 2024; Saboor et al., 2024). Many studies relied heavily on single-source datasets with limited external validation, raising concerns regarding overfitting, dataset bias, and reduced real-world clinical generalizability. Although advanced hybrid and multimodal frameworks improved classification performance, they often introduced higher computational complexity and hardware dependency. Overall, future clinical translation will require multicenter validation, standardized benchmarking, explainable frameworks, and computationally efficient models suitable for real-world neuro-oncological applications.

### 5.6 Limitations

Brain tumor classification studies frequently rely on single-source datasets with limited cross-institutional validation, restricting generalizability across diverse clinical settings. Tumor heterogeneity, multiclass overlap, MRI acquisition variability, and accurate lesion boundary delineation continue to affect classification robustness and segmentation reliability. In addition, extensive preprocessing, augmentation, and hybrid deep learning architectures may increase overfitting risk and computational dependency. Although advanced CNN frameworks achieve high diagnostic performance, limited explainability and hardware-intensive deployment remain major barriers to real-world neuro-oncological applications.

## 6 Cross-disease comparison and emerging insights

Although these disorders differ in pathology and clinical presentation, CNN-based studies for Alzheimer's disease, Parkinson's disease, and brain tumor classification exhibit several common methodological patterns. Table 7 compares the major datasets, imaging modalities, model architectures, validation strategies, strengths, limitations, and clinical translation challenges across the three disorders.

**Table 7:** Cross-disease comparison and methodological quality assessment of CNN-based studies for Alzheimer's disease, Parkinson's disease, and brain tumor classification.

Aspect	Alzheimer's disease	Parkinson's disease	Brain tumor
Cross-disease comparison			
Predominant Datasets	ADNI, OASIS, Kaggle	PPMI, Tao Wu, UK Biobank	Figshare, BraTS, Kaggle
Common Imaging Modalities	MRI, sMRI, PET + MRI	MRI, fMRI, DTI, SPECT	MRI (T1, T1-CE, T2, FLAIR)
Dominant CNN Architectures	ResNet, DenseNet, EfficientNet, CNN-Transformer	Attention CNNs, CNN-LSTM, Res-DenseNet, AMEF-Net	VGG16, DenseNet, YOLO-based CNNs, CNN-LSTM, ParMamba
Validation Strategies	Train-test split, cross-validation, and occasional external validation	Train-test split, cross-validation, and occasional external validation	Train-test split, cross-validation, and occasional external validation
Major Strengths	Early-stage disease classification and progression assessment	Multimodal feature integration and subtle neurodegenerative pattern detection	High tumor classification accuracy and lesion localization
Common Limitations	Dataset bias, class imbalance, limited external validation	Small cohorts, scanner variability, limited external validation	Dataset diversity issues, class imbalance, limited external validation
Clinical Translation Challenges	Generalizability, explainability, and multimodal integration	Data harmonization, reproducibility, and disease-stage differentiation	Scalability, robustness across institutions, and deployment feasibility
Methodological quality assessment			
Independent external validation	3/20 (15%)	4/20 (20%)	2/20 (10%)
Patient-level data separation	4/20 (20%)	8/20 (40%)	10/20 (50%)
Explainable AI (Grad-CAM/SHAP/LIME)	2/20 (10%)	3/20 (15%)	3/20 (15%)
Statistical validation (*p*-value/*t*-test/ANOVA)	2/20 (10%)	5/20 (25%)	0/20 (0%)
Computational efficiency reporting	2/20 (10%) explicit; 18/20 (90%) not reported	16/20 (80%) partial; 4/20 (20%) not reported	4/20 (20%) explicit; 16/20 (80%) partial

To strengthen the comparative analysis, the reviewed studies were further evaluated using a structured methodological quality assessment based on five predefined criteria: independent external validation, patient-level data separation, explainable artificial intelligence (XAI), statistical validation, and computational efficiency reporting. The cross-disease comparison and methodological quality assessment are summarized in Table 7.

The methodological quality assessment demonstrates that relatively few studies performed independent external validation or incorporated explainable artificial intelligence and formal statistical validation. Patient-level data separation was inconsistently reported, increasing the potential risk of data leakage and optimistic performance estimation. Furthermore, computational efficiency was rarely evaluated using deployment-oriented metrics such as parameter counts, FLOPs, inference time, memory consumption, or energy efficiency. These findings indicate that, despite substantial improvements in diagnostic accuracy, methodological rigor and clinical readiness remain important challenges across all three disease categories.

To provide direct quantitative computational benchmarking, representative studies reporting deployment-oriented metrics, including parameter counts, FLOPs, inference time, and hardware configuration, are comparatively summarized in Table 8.

**Table 8:** Quantitative computational benchmarking of representative CNN-based models reported in the reviewed Alzheimer's disease, Parkinson's disease, and brain tumor studies.

References	CNN model	Parameters (*M*)	FLOPs (GFLOPs)	Inference time (ms)	GPU/hardware
Alzheimer's disease					
Awarayi et al. (2024)	Custom CNN (4 Conv + 2 FC)	0.427	NR	NR	NVIDIA GeForce GTX 1060 (8 GB)
Rahman et al. (2025)	3D-CNN	1.091	NR	NR	Intel Core i7, 16 GB RAM
Sorour et al. (2024)	CNN-with-Aug	6.455	NR	NR	Google Colab
Tuncer et al. (2025)	FiboNeXt	9.90	NR	NR	NVIDIA GeForce RTX 2070
El-Assy et al. (2024)	Dual-CNN	57.526	NR	NR	Tesla K80 (Google Colab Pro)
Hassan et al. (2025)	SCCAN	1.241	1.70	NR	NVIDIA GeForce RTX 3090
Asaduzzaman et al. (2025)	ALZENET	4.647	NR	NR	NR
Parkinson's disease					
Camacho et al. (2023)	3D CNN (SFCN-based)	2.96	NR	NR	NR
Sailaja and VenuGopal (2023)	Modified ResNet50V2	NR	0.24	NR	NR
Camacho et al. (2024)	Lightweight 3D CNN	2.98	NR	NR	NVIDIA GeForce RTX 3090
Li et al. (2024)	Improved YOLOv5	3.83	3.3	NR	RTX 3090 (24 GB), Intel Xeon Platinum 8350C
Ding et al. (2025)	AMEF-Net	4.8	NR	NR	NVIDIA A100 (40 GB)
Brain tumor					
Islam et al. (2024)	2D CNN (9-layer), 2D CNN (13-layer), CNN + LSTM	21.59/43.09/3.79	NR	NR	NR
Ilani et al. (2025)	3-Layer CNN /U-Net /VGG19 /InceptionV3 /EfficientNetB4	0.30-31.0	NR	NR	Kaggle (4 × 16 GB GPUs)
Rahman et al. (2024)	GliomaCNN	82.42	NR	NR	NR
Huang et al. (2025)	YOLO-TumorNet	5.66	6.64	NR	NVIDIA RTX 3090 (24 GB VRAM)
Su et al. (2025)	ParMamba	6.47	0.98	NR	NVIDIA RTX 3090 (24 GB VRAM)
Saboor et al. (2024)	A-GRU (CNN-GRU-Attention)	122.07	NR	NR	CPU + GPU (Windows 8, TensorFlow/Keras)
Raza and Iqbal (2025)	Lightweight-CancerNet	NR	NR	3	Intel Core i5-1235U, NVIDIA UHD Graphics 630 Ti (8 GB)
Mao et al. (2025)	Dilated SE-DenseNet	8.08	1.86	NR	NR

GFLOPs, Giga floating point operations; NR, not reported.

The quantitative computational benchmarking presented in Table 8 reveals considerable variability in the computational complexity of CNN-based models across the reviewed studies. Lightweight architectures generally required fewer computational resources, whereas hybrid, attention-based, and deeper models achieved improved diagnostic performance at the expense of increased computational demands and hardware dependency. However, only a limited number of studies reported deployment-oriented computational metrics, limiting objective comparison across models. These findings highlight the need for standardized computational benchmarking to improve reproducibility, fair model comparison, and practical clinical deployment.

Across all three disorders, CNN-based architectures have evolved from conventional networks toward hybrid, attention-based, and multimodal frameworks. However, dataset-specific bias, limited external validation, class imbalance, and explainability remain major barriers to clinical translation. These findings indicate that future progress will depend not only on architectural advances but also on robust validation strategies, data harmonization, and clinically interpretable AI systems.

Reported performance metrics should be interpreted cautiously, as direct comparisons across studies are influenced by differences in datasets, sample sizes, preprocessing pipelines, class definitions, and validation protocols. Consequently, exceptionally high accuracies reported in some studies may not necessarily reflect superior clinical performance or real-world generalizability.

### 6.1 Validation concerns and risk of inflated performance

Several studies across AD, PD, and brain tumor classification reported exceptionally high accuracies exceeding 98%–99% (Slimi et al., 2024; Fathi et al., 2024; Sailaja and VenuGopal, 2023; Mahendran and Visalakshi, 2024; Mithun and Jawhar, 2024; Su et al., 2025). However, many studies relied on relatively small or single-source benchmark datasets such as ADNI, PPMI, Kaggle, Figshare, and REMBRANDT without extensive multicenter or prospective clinical validation. In addition, augmentation, SMOTE/ADASYN balancing, and slice-based MRI analysis were frequently employed to improve performance, which may introduce synthetic-data bias and increase overfitting risk (Slimi et al., 2024; Su et al., 2025). A major methodological concern is the possibility of train-test leakage caused by image-level or slice-level splitting instead of strict subject-wise separation. Several studies reporting near-perfect performance did not clearly specify subject-level partitioning, raising concerns that images or slices from the same patient could appear across training and testing sets. These recurring methodological patterns suggest that exceptionally high accuracies may partly reflect dataset-specific optimization and experimental design choices rather than fully robust clinical generalization.

The cross-disease perspective presented in this review enables the identification of common methodological trends, shared limitations, and clinical translation challenges across AD, PD, and brain tumor classification studies, which are often examined separately in existing reviews.

Many studies reported high diagnostic performance, but limited attention was given to computational complexity, hardware requirements, inference time, and deployment feasibility. Advanced CNN, ensemble, and hybrid architectures may be difficult to implement in real-world clinical settings because of their computational demands. Therefore, future research should emphasize computational efficiency and practical clinical deployment alongside classification accuracy.

## 7 Future directions

**Figure 8:**
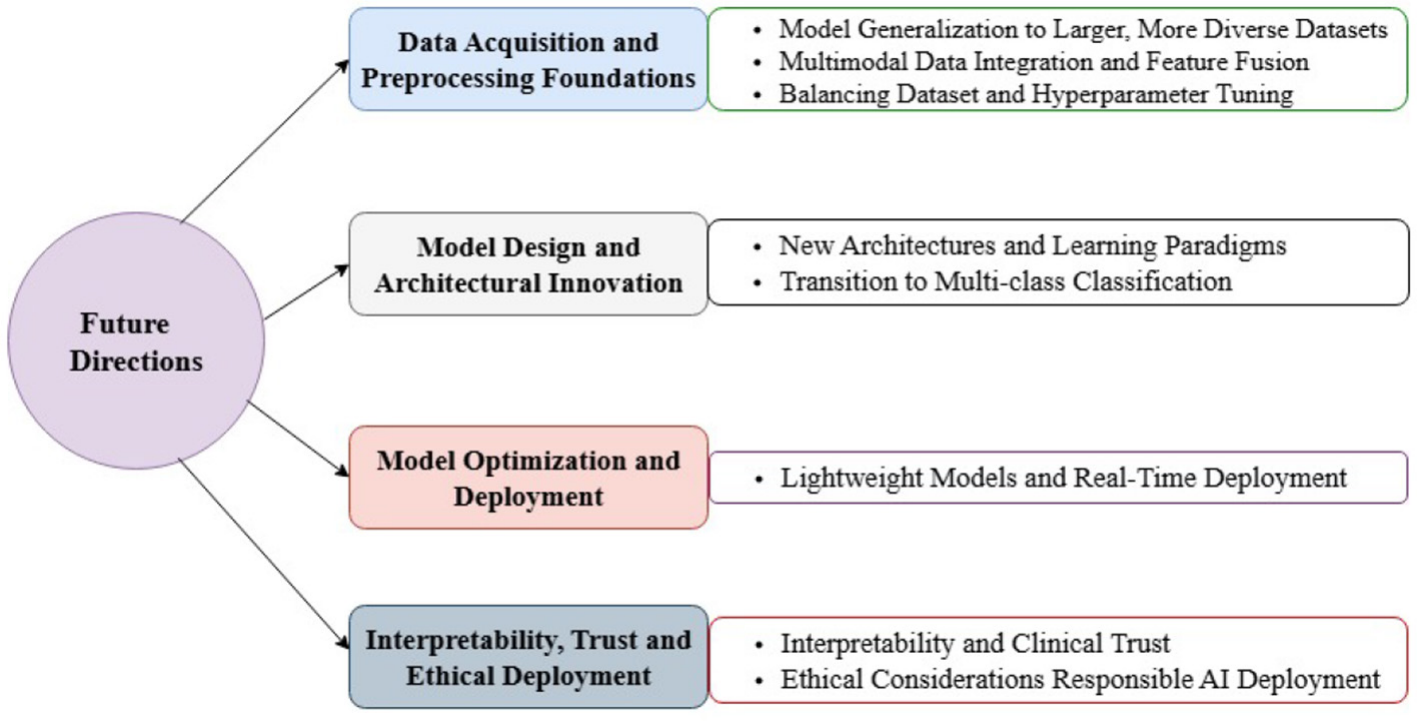
Overview of future directions in CNN-based neurological disorder diagnosis.

As CNN-based methods for diagnosing brain disease progress, future work must focus on some key areas to make the systems clinically applicable, robust, and ethically deployable. These dimensions involve expanding data diversity and preprocessing, new model architectures, real-time optimization, and high priority on interpretability and responsible AI. Resolution of these dimensions allows the next-generation diagnostic systems to be more accurate, generalizable, and trustworthy in the clinical context. Figure 8 depicts the overview of the future directions in CNN-Based Neurological disorder diagnosis.

### 7.1 Data acquisition and preprocessing foundations

- Model generalization to larger, more diverse datasets: it is critical that subsequent studies test and verify CNN-based models on larger, demographically diverse, and multi-center clinical data sets to enhance generalization over real-world cohorts and disease groups (Awarayi et al., 2024; El-Assy et al., 2024).
- Multimodal data integration and feature fusion: the combination of more than one imaging modality, such as MRI, PET, CT, and even wearable sensor information, may provide denser feature sets to classify (Rahman et al., 2025; Kumari et al., 2024). Augmentation with clinical, cognitive, and genetic information may increase diagnosis accuracy further (Tang et al., 2024; Karthigeyan and Rani, 2024).
- Balancing dataset and hyperparameter tuning: minority class identification, hyperparameter adjustment, and data harmonization solutions will be critical for resilience and generalizability (Asaduzzaman et al., 2025; Palakayala and Kuppusamy, 2024b).

### 7.2 Model design and architectural innovation

- New architectures and learning paradigms: hybrid CNN-transformer architectures and nnUNet (Ilani et al., 2025), together with ensemble learning, federated learning, and transfer learning (e.g., MedicalNet) (Rahman et al., 2025), may improve accuracy while maintaining privacy and scalability.
- Transition to multi-class classification: next generation models must be extended from binary classification to multi-class classification to more precisely and effectively diagnose disease phases, especially for complex diseases such as AD and BT (Ragab et al., 2024).

### 7.3 Model optimization and deployment

- Lightweight models and real-time deployment: model optimization techniques such as model pruning, quantization, and lightweight models are essential for real-time clinical deployment (Slimi et al., 2024). Ease of adoption can be facilitated by energy efficiency improvement and cloud support (Agarwal et al., 2024).

### 7.4 Interpretability, trust, and ethical deployment

- Interpretability and clinical trust: some techniques like Grad-CAM, SHAP, LIME, attention, and ROI detection help to create the interpretability of the model and are essential for clinical trust and fairness (Tuncer et al., 2025; Ali et al., 2024b; Majhi et al., 2024).
- Ethical considerations and responsible AI deployment: research should focus on ethical use of AI, including the protection of patient data, minimization of bias, and enhanced transparency and reproducibility. Besides that, transparency in model design, informed consent, and adherence to clinical and legal guidelines are crucial for safe and fair clinical use.

Clinical deployment of CNN-based neuroimaging systems will require rigorous multi-center validation, prospective clinical studies, standardized reporting protocols, and clinician-centered explainability. Further, model calibration, uncertainty estimation, regulatory compliance, and integration with existing clinical workflows should be taken into account for safe, reliable, and effective implementation in clinical practice.

## 8 Conclusion

CNNs have significantly advanced neuroimaging-based diagnosis, providing promising solutions for the identification and classification of AD, PD, and brain tumors. Through automatic and hierarchical feature extraction from MRI images, CNN-based approaches have demonstrated strong performance across a wide range of diagnostic tasks. Recent innovations, including TL, attention modules, 3D CNNs, and hybrid architectures involving transformers or RNNs, have further increased model performance. However, limitations such as dataset bias, limited external and cross-center validation, interpretability issues, and computational burdens continue to restrict widespread clinical adoption. The transition to multimodal fusion, explainable AI, lightweight architectures, and real-time deployment models will be critical for narrowing the gap between research and clinical practice. External validation, ethical considerations, and user-centered tools should take center stage in future work to facilitate scalable, reliable, and patient-centered AI solutions for neurodiagnostics. Optimized and clinically validated CNN-based systems have the potential to support more accurate diagnosis, earlier intervention, and improved clinical decision-making for neurological and neuro-oncological disorders.
